# The ventral premammillary nucleus at the interface of environmental cues and social behaviors

**DOI:** 10.3389/fnins.2025.1589156

**Published:** 2025-04-10

**Authors:** Judney Cley Cavalcante, Fabiano Gomes da Silva, Cristina Sáenz de Miera, Carol Fuzeti Elias

**Affiliations:** ^1^Laboratory of Neuroanatomy, Department of Morphology, Center of Biosciences, Federal University of Rio Grande do Norte, Natal, Brazil; ^2^Department of Molecular and Integrative Physiology, University of Michigan, Ann Arbor, MI, United States; ^3^Department of Obstetrics and Gynecology, University of Michigan, Ann Arbor, MI, United States

**Keywords:** odorants, olfaction, photoperiod, neuroendocrine, hypothalamus, aggression, reproduction

## Abstract

The survival of species heavily depends on social behaviors, which in turn rely on the ability to recognize conspecifics within an appropriate environmental context. These behaviors are regulated by the hypothalamus, which processes signals from both the external environment (such as food availability, photoperiod, and chemical cues from other animals) and the internal state (including sex, estrous cycle stage, nutritional status, and levels of stress). Understanding the brain circuits responsible for specific behaviors in experimental animals is a complex task given the intricate interactions between these factors and the diverse behavioral strategies employed by different species. In this review, we will critically evaluate recent studies focused on the ventral premammillary nucleus (PMv) and discuss findings that reveal the PMv as a key, yet sometimes overlooked, node in integrating external and internal environmental cues. We will examine its structural components, internal connectivity, humoral influences, and associated functions, demonstrating the PMv role in the neural regulation of neuroendocrine responses and social behaviors. While much of the existing research centers on rats and mice as model organisms, we will highlight relevant species differences and include a dedicated section for findings in other species.

## 1 Introduction

The survival of species depends on social behaviors, which rely on conspecific recognition in the appropriate context of external and internal environments. The hypothalamus is the main conductor of these behaviors, integrating signals from the external environment (e.g., availability of food, photoperiod, conspecific odorants) and the internal milieu (e.g., sex, stage of the estrous cycle, nutritional state, and levels of stress). Due to the complex interaction of multiple players and differences in species strategies, many challenges arise in unraveling the relevant brain circuitry for specific behaviors. Defining reproducible behavioral paradigms and physiological correlates in adequate animal models is, therefore, an absolute requirement for data interpretation and for gaining insights into the neural basis of social behaviors.

Over the past 20 years, significant progress in molecular and genetic technologies combined with exquisite imaging approaches have greatly improved the understanding of brain circuits in physiology and behavior (Anderson, 2012; Sternson et al., 2013; Kohl et al., 2018; Bayless et al., 2023). These advancements have facilitated the examination of the neural basis of social bonding, dominance, intraspecies communication, social reward, and sexual and agonistic behaviors. While much has been learned, many gaps remain. Among them are the role of the ventral premammillary nucleus (PMv), the chemical phenotype of defined projections, and the behavioral output in a sex-specific manner.

Compared to other hypothalamic nuclei, such as the medial preoptic nucleus, the ventromedial nucleus of the hypothalamus, and the arcuate nucleus, little is known about the PMv. However, research on this topic has significantly advanced in recent years, revealing that this discrete nucleus plays a crucial role in relaying signals from external environmental cues and the internal milieu to allow for species-specific physiological and behavioral responses.

Here, we will critically discuss the recent literature focused on PMv structural components, intrinsic connectome, and associated functions, highlighting its essential but sometimes neglected role in the neural circuitry of sexual and agonistic behaviors. It should be noted that the bulk of the scientific literature on this topic is focused on rats and mice. Species differences will be highlighted whenever possible, and a separate section will be dedicated to findings in other species.

## 2 Brief historical overview

The premammillary nucleus was first identified by Ramón y Cajal (1911) as “*a nucleus that lies between the mammillary body and the ventromedial nucleus*” in rodents. Situated in the posterior hypothalamus, it was also referred to as the posterior or accessory nucleus of the tuber cinereum. This description implies that Ramón y Cajal was depicting the ventral, not the dorsal, premammillary nucleus.

In 1927, Elisha Gurdjian published a seminal study on the anatomy of the rat diencephalon where they provided a detailed description of the PMv. Gurdjian identified the PMv as a cluster of medium-sized cells situated more rostrally than the dorsal premammillary nucleus, at the lower limits of the hypothalamus. They noted that in its front portion, the PMv contains a group of cells that extend from its upper middle limits in a gentle curve.

“*In a plane farther caudal, the nucleus rounds off, becomes smaller, and occupies a position between the rostral extreme of the lateral mammillary nucleus and the hypothalamic periventricular nucleus. In the plane of the middle third of the mammillary body this nucleus disappears…its dorsal portion disappearing first, while a portion of its ventral part continues caudal into the mammillary body”* (Gurdjian, 1927).

Few years later, Krieg (1932) delineated the structural details of PMv neurons.

“*Golgi impregnations indicate medium or small cells which have from two to four long branching processes which form a loose neuropil in the area occupied by the nucleus*” (Krieg, 1932).

Most of the initial anatomical descriptions were based on rodent brains, but later the PMv was also identified in other species (Kappers et al., 1936; Haymaker et al., 1969).

The role of the PMv in physiology and behavior started to be revealed in studies using electrolytic lesions of hypothalamic nuclei of rats. In search for brain sites associated with metabolic control, Hetherington and Ranson (1940) implicated the PMv as part of the lesioned areas and potentially involved in energy balance.

“*Examination of these lesions has shown them to be very large, but they all have in common extensive bilateral damage to the region occupied by the dorsomedial and ventromedial hypothalamic nuclei, the arcuate nucleus, the fornix, and that portion of the lateral hypothalamic area ventral to it, and probably also the **ventral premammillary nuclei***” (Hetherington and Ranson, 1940).

This is, in fact, a remarkably precise description of the distribution of leptin receptors in the rat hypothalamus (Elmquist et al., 1999). The role of PMv neurons in energy balance, however, is debatable and will be discussed in subsequent sections.

From the late 70s to mid-80s, distinct groups of investigators positioned the PMv as part of the circuitry controlling conspecific behaviors. Using electrolytic lesions and electrochemical stimulation, Taleisnik’s laboratory, working with female rats, proposed that the PMv is part of the vomeronasal circuitry associated with sensing conspecific opposite sex odorants and endocrine reproductive responses. Ovariectomized, estrogen-primed female rats showed increased luteinizing hormone (LH) release when presented with male urine, a response that was blocked by bilateral lesions of the PMv (Beltramino and Taleisnik, 1979, 1983, 1985). Similarly, stimulation of the medial nucleus of the amygdala or the bed nucleus of the stria terminalis induced LH secretion, also absent when the PMv was ablated (Beltramino and Taleisnik, 1978, 1980). They concluded that, in female rats, LH release evoked by male odorants is relayed by the PMv before reaching the final path in the mediobasal hypothalamus (Beltramino and Taleisnik, 1985).

In male rats, studies conducted by Koolhaas group suggested a role for the PMv in agonistic behaviors, i.e., male display of dominance or aggression toward another male of the same species (van den Berg et al., 1983). Male rats with electrolytic lesions of the PMv showed a higher number of attacks and sustained aggressive posture toward a non-lesioned same-sex cage mate.

In the last two decades, with the use of innovative technologies and approaches, consistent and reproducible findings on PMv circuitry and function have supported its role as a key integrative relay for neuroendocrine and behavioral responses.

## 3 Structure and circuitry

In rats and mice, the rostral tip of the PMv coincides with the most rostral aspect of the third ventricle recess, extending toward the caudal end of the ventromedial nucleus of the hypothalamus. The PMv is surrounded medially by the arcuate nucleus and the dorsal tuberomammillary nucleus, ventrally by the ventral limits of the hypothalamus, and dorsally by the fornix, the dorsal premammillary nucleus, and the posterior nucleus of the hypothalamus. Its shape resembles a crescent moon with the concavity turned dorsally and laterally, facing the fornix. Caudally, the PMv has an oval shape with a larger transversal axis, and boundaries lying rostral to the medial mammillary nucleus.

**Figure 1:**
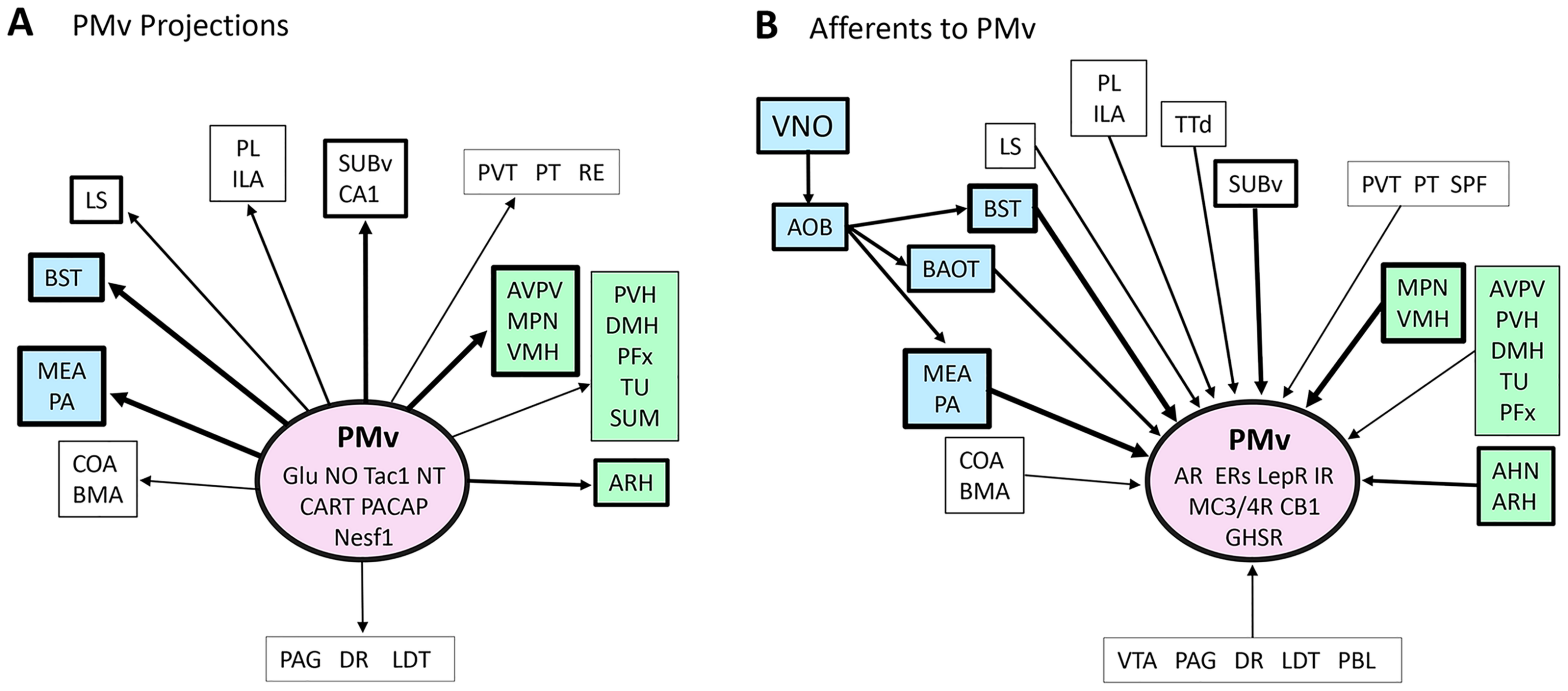
Illustration of the ventral premammillary nucleus (PMv) intrinsic connectome. **(A)** Summary of projections of the rat PMV. **(B)** Summary of the afferent innervation of rats and mice PMv. Vomeronasal relays in blue and hypothalamic output in green. Note the abundance of bidirectional innervation of vomeronasal system nuclei and behavioral control sites. AHN, anterior hypothalamic nucleus; AOB, accessory olfactory bulb; AR, androgen receptor; ARH, arcuate nucleus; AVPV, anteroventral periventricular nucleus; BAOT, bed nucleus of the accessory olfactory tract; BMA, basomedial nucleus of the amygdala; BSTam, anteromedial division of the bed nucleus of the stria terminalis; BSTp, posterior division of the bed nucleus of the stria terminalis; CA1, CA1 field of the hippocampus; CART, cocaine- and amphetamine-regulated transcript; CB1, cannabidiol receptor 1; COA, cortical amygdala; DMH, dorsomedial nucleus of the hypothalamus; DR, dorsal raphe nucleus; Enk, enkephalin; ENT, entorhinal cortex; ERs, estrogen receptors; GHSR, growth hormone secretagogue receptors; Glu, glutamate; GnRH, gonadotrophin release hormone; ILA, infralimbic cortex; IR, insulin receptor; LC, locus coeruleus; LDT, laterodorsal tegmental nucleus; LepR, leptin receptor; LHA, lateral hypothalamic area; LSr, rostral part of the lateral septal nucleus; LSv, ventral part of the lateral septal nucleus; MC3R/4R, melanocortin-3 and 4 receptors; MEA, medial nucleus of the amygdala; MEPO, median preoptic nucleus; MPN, medial preoptic nucleus; Nesf1, nesfatin-1; NO, nitric oxide; NT, neurotensin; PA, posterior nucleus of the amygdala; PAA, piriform-amygdalar area; PACAT, pituitary adenylate cyclase-activating polypeptide; PAG, periaqueductal gray; PBL, lateral division of the parabrachial nucleus; PFx, perifornical area; PH, posterior hypothalamus; PL, prelimbic cortex; PT, paratenial nucleus; PVH, paraventricular nucleus of the hypothalamus; PVT, paraventricular nucleus of the thalamus; RE, reunens nucleus; SPF, subparafascicular parvicellular nucleus; SUBv, ventral subiculum; SUM, supramammillary nucleus; Tac1, tachykinin 1 or substance P; TTd, dorsal part of the taenia tecta; TU, tuberal nucleus; VMH, ventromedial nucleus of the hypothalamus, VMHvl, ventrolateral division of the ventromedial nucleus of the hypothalamus; VNO, vomeronasal organ; VTA, ventral tegmental area. Data based on Canteras et al. (1992), Gautron et al. (2013), and Cavalcante et al. (2014). Abbreviations according to the Allen Brain Atlas, Mouse Brain (https://mouse.brain-map.org/).

The PMv projections were initially described using standard neuroanatomical tracers in rats (Canteras et al., 1992; Cavalcante et al., 2014) and mice (Gautron et al., 2013) (Figure 1A). A predominant innervation of the forebrain as rostral as the prelimbic cortex was noticed. Low to moderate inputs were observed in the infralimbic cortex, ventral subiculum, CA1 field, lateral septum, and medial thalamic nuclei such as the paraventricular, paratenial, reuniens, and mediodorsal (Canteras et al., 1992). The main targets, however, were nuclei of the hypothalamus, amygdala, and bed nucleus of the stria terminalis, components of the accessory olfactory pathway or vomeronasal system (VNS) (Scalia and Winans, 1975; Canteras et al., 1992; Boehm, 2006; Gautron et al., 2013).

The VNS comprises a group of forebrain nuclei indirectly innervated by the vomeronasal organ (VNO), relayed by the accessory olfactory bulb. They receive and process olfactory information related to conspecific (socially relevant) and heterospecific (socially non-relevant) interactions (Halpern and Martínez-Marcos, 2003). The VNS encompasses the accessory olfactory nucleus, and divisions of the bed nucleus of the stria terminalis, the medial and posterior nuclei of the amygdala, and subdivisions of the cortical amygdala (Scalia and Winans, 1975).

The PMv is also part of the VNS, comprising the third relay station of the vomeronasal network (Segovia and Guillamón, 1993; Guillamón and Segovia, 1997). It densely projects to the arcuate nucleus and the ventrolateral subdivision of the ventromedial nucleus of the hypothalamus, key components of neuroendocrine and behavioral circuitry, respectively (Canteras et al., 1992; Gautron et al., 2013).

The PMv innervates the medial preoptic area, the anteroventral periventricular nucleus (AVPV), and the gonadotropin-releasing hormone (GnRH) neurons, the final neural output in the neuroendocrine reproductive axis (Rondini et al., 2004; Boehm et al., 2005; Yoon et al., 2005; Leshan et al., 2009; Louis et al., 2011). The AVPV is a sexually dimorphic site (bigger in females), fundamental for the LH surge at mid-cycle (Wiegand et al., 1978; Wang et al., 2019). Together, a simple model would predict that neurons of the PMv relay odorant cues from distinct sources to AVPV and GnRH neurons modulating neuroendocrine responses.

The afferents to the PMv are mostly reciprocal (Figure 1B), i.e., they originate from VNS and gonadal steroid-sensitive nuclei in rats of both sexes (Nakano et al., 1997; Cavalcante et al., 2014). Outside the VNS, the ventral subiculum and the ventral subdivision of the lateral septum extend dense to moderate projections to the PMv (Canteras et al., 1992; Cavalcante et al., 2014).

Although the general PMv “connectome” is thoroughly described in rats, afferent inputs in mice are lacking and the chemical identity of these pathways is understudied. The PMv receives projections from calcitonin gene-related peptide (CGRP) neurons of the preoptic area (Spratt and Herbison, 2002) and from urocortin 3 neurons of the medial nucleus of the amygdala and perifornical area (Cavalcante et al., 2006b), but the functional relevance of these projections is unknown.

## 4 Chemical identity

The PMv neurons densely express the fast-acting excitatory neurotransmitter glutamate (Ziegler et al., 2002; Lin et al., 2003; Donato et al., 2011). A subset of these neurons also produces nitric oxide (NO), a gaseous, highly soluble, and membrane-permeable neurotransmitter (Vincent and Kimura, 1992; Rodrigo et al., 1994; Gotti et al., 2005). Due to its gaseous nature, NO is primarily detected via labeling of synthetizing or metabolizing enzymes, such as NO synthase (neuronal or nNOS) and nicotinamide adenine dinucleotide phosphate (NADPH)-diaphorase (Hope et al., 1991). NO amplifies glutamatergic neurotransmission via actions in NMDA receptors and induces cellular responses via dephosphorylation of guanosine-triphosphate to cyclic guanosine-monophosphate (cGMP) (Wang and Robinson, 1997; Smolenski et al., 1998).

Data on the neuropeptidergic makeup of PMv is limited. In rats, substance P, a member of the tachykinin family (a.k.a. Tac1), is abundant with higher expression in males (Warden and Young, 1988; Larsen, 1992). Corticotropin-releasing factor (CRF), vasoactive intestinal polypeptide (VIP), cocaine- and amphetamine-regulated transcript (CART), enkephalin, neurotensin, and nesfatin-1 are also observed but at lower levels (Zoli et al., 1993; Lantos et al., 1995; Papp et al., 2025). In mice, Tac1 and neurotensin distribution are similar to that observed in rats, whereas VIP, CRF, and CART expression are lower or undetectable (Wamsley et al., 1980; Shimada et al., 1987; Zoli et al., 1993; Douglass et al., 1995; Lantos et al., 1995). Dense expression of pituitary adenylate cyclase-activating polypeptide (PACAP) has been reported in the mouse PMv (Ross et al., 2018).

Whether these neuropeptides are part of specific circuitry is unclear. CART and NO are coexpressed in PMv neurons (Donato et al., 2010a), but the functional significance of this finding is unknown. CART was first described as a neuropeptide in the rat striatum modulated by psychostimulants (Douglass et al., 1995). It was later observed throughout the central nervous system and has been implicated in a variety of physiological processes, including feeding, stress response, and reproduction (Couceyro et al., 1997; Koylu et al., 1997, 1998; Rondini et al., 2004; Venancio et al., 2017; Ahmadian-Moghadam et al., 2018). In rats, PMv CART neurons project to the AVPV, and CART immunoreactive fibers are in close apposition with GnRH neurons (Leslie et al., 2001; Rondini et al., 2004). In the mouse, projections of PMv CART neurons are yet to be defined.

Humans and mice with loss-of-function mutations in nNOS (encoded by *NOS1* and *Nos1* genes, respectively) are infertile and global inhibition or deletion of NO production precludes leptin-induced LH secretion (Yu et al., 1997; Gyurko et al., 2002; Bellefontaine et al., 2014; Chachlaki et al., 2022). Of note, androgen receptors (AR) are highly coexpressed in NO/CART neurons of the rat PMv (Yokosuka et al., 1997) and NO/leptin receptor neurons of the mouse PMv (Donato et al., 2010a; Leshan et al., 2012; Cara et al., 2020).

A fascinating aspect of the PMv is the abundance and variety of receptors for internal environmental cues. It expresses virtually all gonadal steroid nuclear receptors, e.g., estrogen receptor alpha and beta, progesterone receptor, and AR, with particularly dense levels of AR (Simerly et al., 1990; Shughrue and Merchenthaler, 2001; Cara et al., 2021). Receptors for leptin (LepR), ghrelin, insulin, calcitonin, uroguanylin, prolactin, and growth hormone are also observed in PMv neurons (Elmquist et al., 1998; Elias et al., 2000; Zigman et al., 2006; Scott et al., 2009; Garcia-Galiano et al., 2017; Merlino et al., 2019; Silveira et al., 2019).

The PMv densely expresses receptors for peptides and unconventional neurotransmitters associated with whole-body homeostasis such as melanocortin-3 and-4, neuropeptide Y type 1, orexin, fatty acids (CD36), opioids, and endocannabinoids (Brown et al., 1996; Marcus et al., 2001; Liu et al., 2003; Wittmann et al., 2007; Glezer et al., 2009; Bedenbaugh et al., 2022). Little is known about their interplay, but a few findings are of interest. A subset of PMv LepR neurons express insulin receptors, but no coexpression between LepR and melanocortin-4 receptors or leptin-induced STAT-3 and nesfatin-1 was identified, indicating a dissociation of metabolic pathways within this site (Gautron et al., 2013; Garcia-Galiano et al., 2017; Papp et al., 2025).

Dopamine transporter (DAT, *Slc6a3* gene), a membrane protein involved in dopamine reuptake at presynaptic terminals, is also expressed in PMv neurons (Meister and Elde, 1993). These DAT neurons are intriguing because they exhibit very low or undetectable levels of tyrosine hydroxylase (TH) and seem unable to release dopamine (Soden et al., 2016; Yip et al., 2018). Nevertheless, PMv DAT neurons contain other components of the dopamine regulatory pathway, including dopamine decarboxylase (*Ddc* or *Aadc* gene), vesicular monoamine transporter 2 (*Vmat2* or *Slc18a2* gene) and guanylyl cyclase 2C (*Gucy2c* gene) (Merlino et al., 2019; Soden et al., 2016; Han et al., 2020). The role of PMv DAT neurons has been incompletely defined and will be discussed in another section.

## 5 Relevance for social behaviors

**Figure 2:**
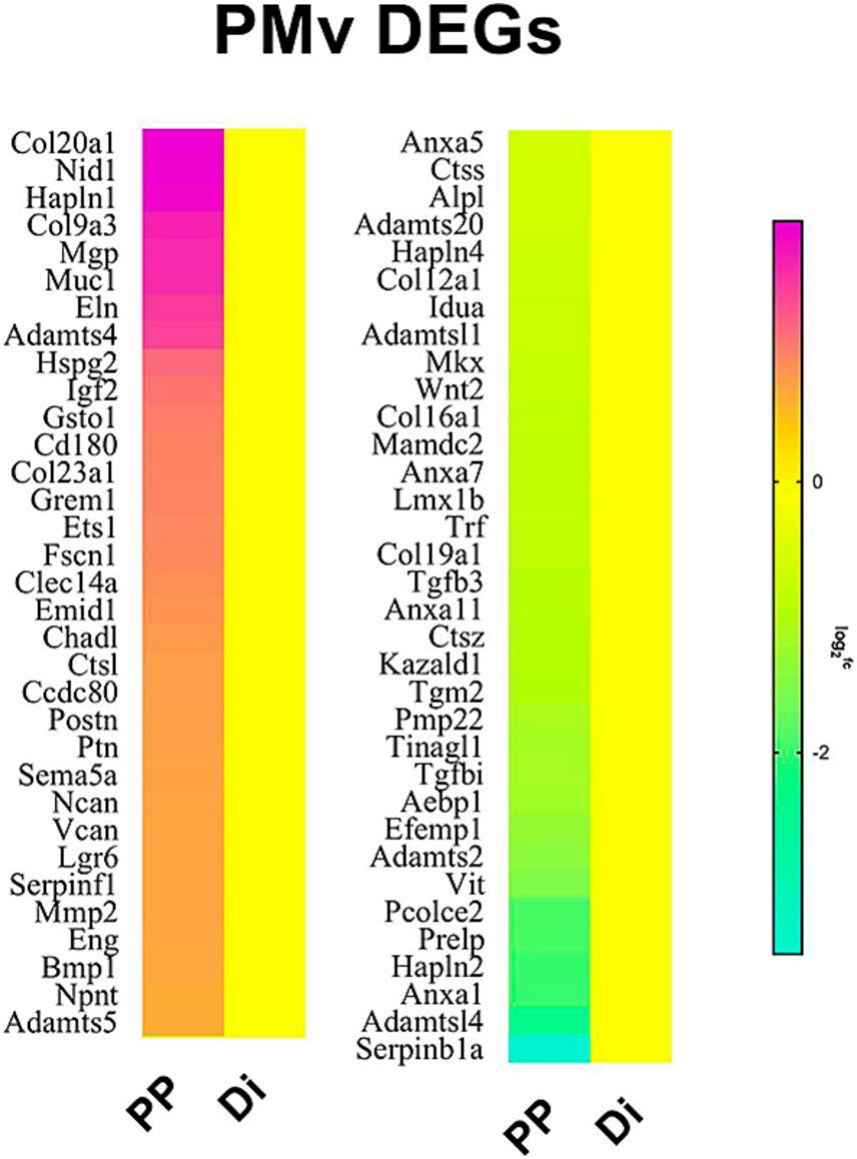
Differentially expressed genes associated with extracellular matrix in the ventral premammillary nucleus (PMv) of prepubertal (PP) vs. diestrous (Di) female mice. Data obtained from Han et al. (2020).

In the last decades, it has become clear that the PMv of rodents plays a crucial role in the integration of external (olfactory in most mammals) and internal environmental cues to allow for adequate behavioral responses. These responses are mostly associated with conspecific recognition and reproductive strategies in both sexes and are stablished during pubertal transition (Sisk and Zehr, 2005; Wommack and Delville, 2007). Using comparative bulk RNA sequencing of PMv from prepubertal and adult female mice, we showed that differentially expressed genes were highly enriched for extracellular matrix (Han et al., 2020) (Figure 2). In fact, abundant perineuronal nets (PNN), a specialized form of extracellular matrix, are observed in PMv neurons with higher levels in males (Ciccarelli et al., 2021). These glycoproteins surround the soma of selected subtypes of neurons and appear to have a role in restraining neuronal structural plasticity (Sorg et al., 2016). Rather than “lattice-like,” the PNN in PMv neurons has a semi-organized “cotton wool-like” aspect, typical of that found in glutamatergic neurons. This structure indicates a more permissive extracellular environment for synaptic and dendritic remodeling (Fawcett et al., 2019; Ciccarelli et al., 2021). However, abundant PNN in PMv neurons also suggest limited plasticity, at least during adult life in a sex-specific manner (Sorg et al., 2016; Ciccarelli et al., 2021). In females, studies have emphasized a role in the neuroendocrine reproductive axis, while in males, the PMv has been associated with agonistic behaviors.

### 5.1 Integrating olfactory cues and gonadal steroids milieu

**Figure 3:**
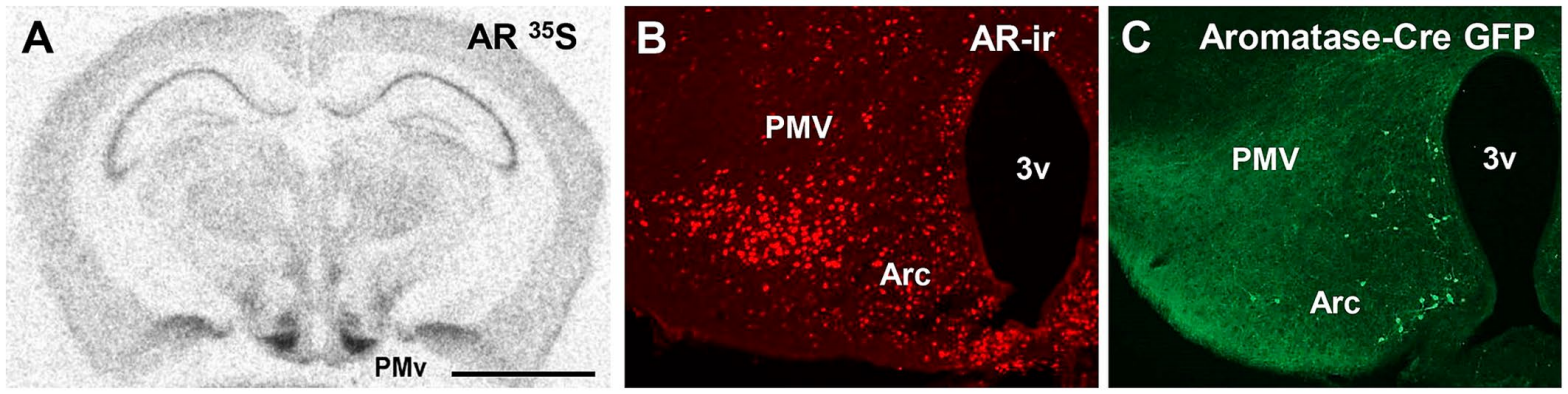
The ventral premammillary nucleus (PMv) expresses a dense collection of sex steroid receptors. **(A)** Bright field image showing the dense expression of androgen receptors (AR) in the mouse PMv. **(B)** Fluorescent image showing AR immunoreactivity in the PMv. **(C)** Fluorescent image showing lack of aromatase expression in the mouse PMv using aromatase-cre induced GFP. The aromatase-Cre mouse model was kindly provided by Dr. Nirao Shah (Stanford University). 3v, third ventricle; AR, androgen receptor; Arc, arcuate nucleus; GFP, green fluorescent protein; PMV, ventral premammillary nucleus. Scale bar: **(A)** 2 mm, **(B,C)** 400 μm.

The PMv directly senses the individual’s reproductive status via androgens and estrogen receptors (Simerly et al., 1990; Shughrue and Merchenthaler, 2001; Merchenthaler et al., 2004; Cara et al., 2021). PMv neurons are, however, devoid of aromatase (Figure 3), indicating that circulating androgens affect male and female PMv neurons independent of aromatization to estradiol. The PMv also expresses Fos immunoreactivity (Fos-ir) in response to mating, exposure to opposite-sex odorants, and agonistic encounters (Kollack-Walker and Newman, 1995; Coolen et al., 1996; Yokosuka et al., 1999; Veening et al., 2005; Cavalcante et al., 2006a; Donato et al., 2010b; Donato et al., 2013; Lin et al., 2011). To what extent these neurons overlap and the relevant circuitry of independent subpopulations of neurons are not completely defined.

In male rats, exposure to opposite-sex odors induces Fos-ir in NO and CART neurons and increases *Cartpt* expression in the PMv (Cavalcante et al., 2006a; Donato et al., 2010a). As mentioned in a previous section, PMv CART neurons innervate areas associated with reproductive neuroendocrine control (Rondini et al., 2004). In fact, lesions of the female PMv temporarily disrupt estrous cycles, alter cyclic changes in circulating gonadal steroids and gonadotropins, and disrupt the dynamic changes in GnRH expression and neuronal activation in the proestrous day. It also decreased female receptivity (lordosis) accompanied by a lower number of mounts and ejaculation rate of an intact male breeder (Donato et al., 2009; Donato et al., 2013). Together, these findings suggest that PMv CART neurons convey information on sexually relevant odorants to the reproductive axis, modulating neuroendocrine and behavioral responses.

PMv CART neurons also coexpress LepR (Elias et al., 2000, 2001). In mice, PMv LepR neurons respond to conspecific and heterospecific (predators) odorants (Leshan et al., 2009; Yuan et al., 2024). Because leptin signals energy status, we evaluated if responses to olfactory cues were modified in mice under negative energy balance. No difference in the number of Fos-ir neurons in an experimental paradigm of acute (overnight) fasting was observed (Donato et al., 2010b). However, hypoglycemia reduced odor-induced Fos-ir in nesfatin-1 non-LepR PMv neurons (Papp et al., 2025).

Socially relevant odors induce physiological arousal in rodents and humans (Wang et al., 2012; Lübke and Pause, 2015). Using LepR neurons as a genetic tool (LepR-Cre mouse model), studies found that remote activation of PMv LepR neurons increased wakefulness, whereas inhibition of those neurons precluded odorant effect on sleep/wake pattern and duration (Yuan et al., 2024). Together, these findings indicate that the PMv also relays environmental information to areas controlling sleep/wake cycles. Independent studies are necessary to reproduce these findings and define the projections associated with this compelling observation.

Approximately half of PMv LepR neurons are sensitive to androgens in male and 35% in female mice (Cara et al., 2020, 2021). Lack of AR in LepR neurons (LepR^ΔAR^ mice) induced no changes in food intake, fasting blood glucose, sexual maturation, or fertility (Cara et al., 2020). However, male LepR^ΔAR^ mice exhibited increased ambulatory activity, higher basal testosterone levels, and exaggerated response to negative feedback actions of sex steroids, i.e., increased LH levels following orchidectomy. Because high testosterone and LH levels are hallmarks of polycystic ovary syndrome (PCOS), we assessed if AR in LepR neurons plays a role in the neuroendocrine dysregulation of hyperandrogenemia-induced PCOS-like phenotype in mice. Notably, the lack of AR in LepR neurons restored estrous cycles in prenatally androgenized female mice (Cara et al., 2023).

### 5.2 Integrating metabolic cues and the reproductive neuroendocrine axis

PMv neurons express a variety of receptors for metabolic cues. Of those, LepR is the most well-studied to date. Leptin is an adipocyte-derived hormone that signals whole-body fat content, and the amount of energy stored. Mice lacking leptin (ob/ob, *Lep* gene) or leptin receptor (db/db, *Lepr* gene) are obese, infertile, and have hypogonadism, among other disturbances (Barash et al., 1996; Chehab et al., 1996; Casanueva and Dieguez, 1999; Elias and Purohit, 2013). Leptin administration to leptin-deficient mice increases LH levels, a response that is ineffective when the PMv is bilaterally ablated (Donato et al., 2011).

Food restriction in wild-type rats and mice decreases circulating levels of leptin and inhibits the reproductive axis (Marsteller and Lynch, 1987; Ahima et al., 1996). Likewise, significant weight loss or energy deprivation in women reduces LH secretion, leading to amenorrhea (Pirke et al., 1985; Bronson and Manning, 1991; Loucks et al., 1998; Warren et al., 1999). Leptin treatment partially restores the levels of gonadal steroids and gonadotropins, improving LH pulse frequency and ovulation in rodents and humans under negative energy balance (Ahima et al., 1996; Gruaz et al., 1998; Nagatani et al., 1998; Schneider et al., 1998). This effect is relayed, at least in part, by the PMv as the increase in LH by acute leptin treatment was prevented in PMv-lesioned rats (Donato et al., 2009). Moreover, in mice, activation of leptin-responsive PMv neurons elevates LH levels even in a neutral energy balance (Sáenz de Miera et al., 2024).

Excessive leanness in prepubertal girls delays sexual maturation, while an excess of body fat has been associated with early onset of puberty (Frisch, 1985; Dunger et al., 2005; Ahmed et al., 2009; Rosenfield et al., 2009; Biro et al., 2010; Burt Solorzano and McCartney, 2010; Reinehr and Roth, 2019; Anderson et al., 2024). Sexual maturation is a complex process that involves a variety of regulatory systems and hormones. Leptin is one of them, exerting a facilitatory role in the onset of puberty (Ahima et al., 1997; Elias, 2012; Anderson et al., 2024). Humans with a loss-of-function mutation in *LEP* gene are infertile, remaining in a prepubertal state unless leptin is replaced (Farooqi et al., 1999). Short-term leptin treatment of leptin-deficient mice precipitates puberty onset, an effect that is also disrupted in PMv-lesioned mice (Cheung et al., 1997; Donato et al., 2011; Han et al., 2020).

The role of PMv LepR neurons in leptin action in sexual maturation is strengthened by studies using genetic manipulation or, more specifically, conditional re-expression of *Lepr* gene in the PMv of LepR null mice. This strategy allows for the evaluation of the actions of LepR in targeted nuclei in the absence of leptin signaling elsewhere. Unilateral re-expression of endogenous LepR in the PMv of LepR null female mice induced puberty and improved fertility with no change in body weight and food intake (Donato et al., 2011). However, this manipulation did not result in successful pregnancies at term. Most pregnant females showed resorptions or delivered pups with malformations (Mahany et al., 2018). This poor pregnancy outcome following restoration of LepR in PMv neurons may reflect the mouse adiposity and insulin resistance and/or lack of leptin signaling in key target cells (Zuure et al., 2013; Egan et al., 2017; Childs et al., 2021).

About half of all PMv neurons express LepR and virtually all PMv LepR neurons are glutamatergic (Elmquist et al., 1997; Elias et al., 2000, 2001; Donato et al., 2009; Leshan et al., 2009; Caron et al., 2010; Zuure et al., 2013). Whereas lack of LepR in glutamatergic neurons did not disrupt the reproductive function (Zuure et al., 2013; Martin et al., 2014), female mice lacking vesicular glutamate transporter 2 (Vglut2) in LepR neurons developed late-onset obesity, altered pubertal maturation, disrupted estrous cycles, and mild subfertility (Sáenz de Miera et al., 2024). Because LepR and Vglut2 are coexpressed in other cells, we used the LepR null mice to assess the role of PMv in those effects. Endogenous re-expression of LepR in PMv neurons restores sexual maturation in otherwise prepubertal LepR null mice (Donato et al., 2011; Mahany et al., 2018). This response is blocked in mice with deletion of Vglut2 selectively in PMv neurons (Sáenz de Miera et al., 2024). Together, these findings indicate that other metabolic cues may overcome the lack of leptin signaling in glutamatergic neurons, but the specific reproductive role of leptin in the PMv requires glutamate neurotransmission.

Leptin depolarizes 75% of the PMv LepR neurons through PI3K-induced activation of TRPC channels and hyperpolarized the remaining 25% through an ATP-gated potassium channel (Leshan et al., 2009; Williams et al., 2011). Of note, about 70% of PMv LepR neurons coexpress PACAP (Ross et al., 2018). Lack of PACAP in PMv neurons (PMv^PACAP^ KO mouse) induced marked disruption of the reproductive function. PMv^PACAP^ KO female mice exhibited longer estrous cycles, decreased capacity to produce a litter after mating, and increased latency to pregnancy. These effects appear to be partially attained via the downstream innervation of kisspeptin neurons (Ross et al., 2018), a crucial component of the reproductive neuroendocrine axis. Inactivating mutations in the kisspeptin (*Kiss1*) gene or its receptor (*Kiss1r*) result in congenital hypogonadotropic hypogonadism and failure to progress through puberty in humans and mice (de Roux et al., 2003; Seminara et al., 2003; Topaloglu et al., 2012).

A subpopulation of PMv LepR neurons coexpress DAT, though their role in neuroendocrine regulation remains unclear. *Lep^ob^* females exhibit lower PMv *Slc6a3* (DAT) expression, which is restored after short-term leptin treatment-induced puberty onset (Sáenz De Miera et al., 2025). The expression of PMv-DAT is higher in prepubertal mice and adult females compared to adult males. In females, PMv-DAT neurons project densely to AVPV Kiss1 neurons, which are part of a sexually dimorphic circuitry associated with mid-cycle LH surge (Wang et al., 2019). The significance of the sex differences in DAT expression and neuronal projections is currently under investigation.

All in all, it seems reasonable to assume that PMv LepR neurons synapse onto GnRH and kisspeptin neurons using glutamate, PACAP, CART, and NO as neurotransmitters (Leshan and Pfaff, 2014; Hill and Elias, 2018; Anderson et al., 2024). Those neurons relay olfactory and humoral signals required for the onset of puberty and reproductive function in adult mice. It should be noted that these physiological effects are sexually dimorphic, i.e., they are observed mainly in females. In fact, a small proportion of leptin-signaling deficient male mice are fertile (Lane and Dickie, 1954; Elias and Purohit, 2013), and leptin action in the testis seems to have a key action linking metabolism and reproductive function (Tena-Sempere and Barreiro, 2002; Borges et al., 2017). The relevance of PMv circuitry for males has been associated with behavioral strategies for successful reproduction, not neuroendocrine responses.

### 5.3 Aggressive behaviors: territoriality and protection of the offspring

Several laboratories have shown that agonistic encounters activate (i.e., induce Fos-ir) PMv neurons of rats, mice, and hamsters (Kollack-Walker and Newman, 1995; Veening et al., 2005; Lin et al., 2011). Using genetic tools in mouse models, studies have identified the PMv DAT neurons as key players in inter-male aggression. PMv-DAT neurons are glutamatergic and in male mice they show Fos-ir in the presence of conspecifics of both sexes (Soden et al., 2016). These neurons are more responsive to either same-sex odorants or intruders when compared to female urine, pups, or male rats (Soden et al., 2016; Chen et al., 2020). Inhibition or ablation of PMv-DAT neurons abolished male mice’s response to male urine and diminished the aggressiveness toward an intruder, with no effect on sexual behavior (Soden et al., 2016; Stagkourakis et al., 2018; Chen et al., 2020).

The role of PMv-DAT neurons in agonistic behaviors, however, is marked by individual nuances. Optogenetic stimulation of PMv-DAT neurons of aggressor resident mice increased attack toward intact or orchidectomized male and female intruders. In non-aggressor mice, activation of PMv DAT neurons increased exploratory behavior (Stagkourakis et al., 2018).

The progesterone receptor (PR) is also expressed in PMv neurons and has been utilized as a genetic tool to assess the PMv role in response to odorants. Activation of the PMv PR neurons promoted, and inhibition suppressed, intermale aggression (Itakura et al., 2022). In males, PMv PR neurons are activated by the presence of male and female mice and urine odorants. These neurons respond to 53AF, a low-molecular-weight fraction of male urine that activates a specific VNO receptor (Vmn2r53) associated with sensing aggression-promoting urinary odorants (Chamero et al., 2011; Itakura et al., 2022). Ablation of Vmn2r53 eliminated 53AF’s effect on PMv PR neurons and diminished the response of these neurons to male mice urine, while the response to female or orchidectomized male urine was intact (Itakura et al., 2022).

Female rodents can also exhibit aggressive behaviors, though these behaviors are less common and less intense than in males. Heightened aggression is often observed when females are protecting their offspring. This behavior, known as maternal aggression, is driven by olfactory cues and aims to defend their young from potential threats (Ferreira and Hansen, 1986; de Almeida et al., 2014). Increased Fos-ir in the rat PMv has been reported following episodes of maternal aggression, and maternal aggression is reduced after PMv ablation (Motta et al., 2013). PMv-DAT neurons are hyperexcited in aggressive compared to non-aggressive dams and virgin mice (Stagkourakis et al., 2024). Optogenetic stimulation of these neurons enhances aggressive behavior and reduces maternal care in lactating dams. This stimulation does not trigger aggressive behavior in virgins, although it does lead to increased investigation of a conspecific.

Overall, the PMv plays a key role in various forms of aggressive behavior, including intermale and maternal aggression. The neural circuitry underlying these behaviors is not completely defined, but in both cases, the ventrolateral subdivision of the ventromedial nucleus of the hypothalamus appears to be the main downstream effector (Hashikawa et al., 2017; Georgescu et al., 2022). PMv neurons relay odorant signals to the ventrolateral ventromedial nucleus of the hypothalamus in both sexes, modulating the transient nature of aggression through the sensing of gonadal or placental hormones.

## 6 Other potential functions

Although the PMv neurons express receptors for hormones and neuropeptides related to metabolic regulation, their role in energy balance has been contentious. Lesion of the PMv did not affect body weight, body composition, mean food intake, and circulating leptin levels in rodents (Donato et al., 2009; Donato et al., 2011). Ablation of PMv neurons, re-expression of LepR in LepR null mice, or disruption of PACAP in LepR neurons does not affect body weight (Donato et al., 2009; Donato et al., 2011; Ross et al., 2018). Few studies, however, have suggested that the PMv has a minor role in energy balance. For example, the administration of ghrelin into the PMv altered the respiratory exchange ratio and glucose homeostasis in the absence of changes in caloric intake or fat mass (Hyland et al., 2021). Disruption of leptin signaling in both PMv and the lateral hypothalamic area resulted in a higher respiratory exchange ratio and altered glycemic control with no effect on either body weight, food intake, energy expenditure, or locomotor activity (Denroche et al., 2016).

In the 80s, two studies from Shiraishi and Mager implicated the PMv in the modulation of body temperature (Shiraishi and Mager, 1980a, 1980b). After these initial publications, little evidence was collected reproducing the findings. Nevertheless, recent studies from a different group suggested that orexin-responsive neurons in the PMv are associated with motion sickness-induced hypothermia (Pan et al., 2024).

As expected in all scientific findings, the role of PMv in respiratory exchange ratio, glucose homeostasis, and thermogenesis requires independent corroboration.

## 7 The PMv in other mammals and in birds

As mentioned, the bulk of scientific literature on PMv has focused on rats and mice as model organisms. However, examination of the PMv in other species has revealed insights into its potential role based on consistent hypothalamic location and aspects of its structural and chemical phenotype. Notably, PMv neurons in several mammal and bird species of seasonal reproduction are responsive to photoperiodic (perceived day length) related cues instead of odorants.

### 7.1 PMv in other mammals

The PMv in other rodent species, from hamsters to rock cavy (*Kerodon rupestris*, a crepuscular rodent), has comparable structural features (Reis et al., 2018). PMv neurons of Siberian hamsters (*Phodopus sungorus*) exhibit vasopressin binding in a sex-dependent manner, with higher levels in males. No photoperiodic variation has been described for vasopressin binding, indicating no changes in vasopressin receptors’ expression across seasons, as opposed to what was observed in other hypothalamic regions (Dubois-Dauphin et al., 1991). The PMv of Siberian hamsters (*Phodopus sugorus*) also expresses thyroid hormone receptor beta (*Thrb*) (Barrett et al., 2007), whereas in Syrian hamsters (*Mesocricetus auratus*), the PMv coexpresses AR and nNOS as well as thyroid hormone receptors alpha (*Thra*) (Hadeishi and Wood, 1996; Jansen et al., 1997).

The PMv of the Brazilian opossum (*Marmosops paulensis*), a nocturnal marsupial, expresses estrogen receptors and shows structural similarities to the rodent PMv (Fox et al., 1991).

Estrogen receptors, thyroid hormone receptor alpha, as well as CART and nNOS, were also observed in the sheep PMv (Jansen et al., 1997; Sliwowska et al., 2004). In ewes, the PMv is a small nucleus located within a larger area defined as the premammillary region (PMH) that also encompasses the caudal arcuate and tuberomammillary nuclei (Sliwowska et al., 2004). Melatonin binding and high-affinity melatonin receptor MT1 (previously known as MTNR1A) were described in the PMH, in a region likely corresponding to ewes’ PMv (Malpaux et al., 1998; Migaud et al., 2005).

In mammals, melatonin is the main hormone that links photoperiodic cues to reproductive function. Melatonin micro-implants placed in the PMH of ewes housed under a long photoperiod (i.e., a non-breeding season) induced LH release (Malpaux et al., 1998). It is worth noting, however, that the primary site of melatonin action in the seasonal control of reproduction is the pituitary gland *pars tuberalis* (Dardente et al., 2019). Moreover, the arcuate nucleus and tanycytes (cells lining the ventral wall of the third ventricle), both located in the adjacencies of the PMv, have also been involved in the seasonal control of reproduction. Whether the ewes’ PMv is a melatonin target for seasonal reproduction requires further investigation.

The premammillary area is poorly defined in primate brains. Using autoradiography, a dense dihydrotestosterone (DHT) radiolabeled signal was described in the PMv of rhesus monkeys (Michael and Rees, 1982). However, no illustrations of this observation are available, making their findings difficult to interpret or compare with other species.

In the cynomolgus monkey (*Macaca fascicularis*), a premammillary nucleus (PM) was described rostral to mammillary nuclei in a ventral position, adjacent to the ventral borders of the hypothalamus, where neurons expressing AR immunoreactivity were described (Veazey et al., 1982; Michael et al., 1995). We found a similar PM localization in the common marmoset (*Callithrix jacchus*) and the capuchin monkey (*Cebus apella*) using CART immunoreactivity and NADPH diaphorase (Cavalcante et al., 2011). Due to challenges in defining the borders of the PMv, we designated this area as the PM to align with the nomenclature used for the cynomolgus monkey. Of note, when analyzing the distribution of PNN in the reproductive neural circuit of the common marmoset, Ciccarelli and co-workers identified this hypothalamic region as the PMv (Ciccarelli et al., 2021).

### 7.2 PMv in birds

In birds, the region described as the nucleus inferior, also known as the “nucleus premammillaris medialis” (PMM), appears to have homologies with the mammalian PMv (Crosby and Showers, 1969). It is located immediately dorsal to the nucleus infundibularis, the rodent arcuate nucleus, at the most caudal levels of the hypothalamus. The PMM expresses CART in songbirds (Singh et al., 2016, 2020), shows high-affinity binding to 3H-estradiol in ring doves (*Streptopelia risoria*) (Martinez-Vargas et al., 1976), and expresses metabolic markers such as NPY in chickens (Kameda et al., 2001). Strong projections from the PMM to the lateral septum have been described in chickens (*Gallus domesticus*) (Montagnese et al., 2008).

In other avian species, the PMM is described in the caudal hypothalamus, above the third ventricle recess (Kang et al., 2007; Moore et al., 2018). It is a dopaminergic area that expresses tyrosine hydroxylase (TH), the rate-limiting enzyme for dopamine production (Kang et al., 2007; Haas et al., 2017; Maney et al., 2020). In turkeys (*Meleagris gallopavo*), the PMM expresses serotonin receptors 5-HT2C and 5-HT1A and shows strong Fos-ir in response to a 5-HT2C agonist (Bakken et al., 2014). Retrograde labeling has demonstrated serotonergic projections to the PMM and close 5-HT immunoreactive appositions to PMM TH cells (Kang et al., 2009).

The ability of avian PMM neurons to perceive light and photoperiodic changes has suggested a role in the seasonal control of reproduction. In turkey hens, the dopaminergic neurons of the PMM express tryptophan hydroxylase 1 (TPH1) and arylalkylamine N-acetyltransferase (AANAT), both key enzymes of melatonin synthesis (Kang et al., 2007). *TH* and *TPH1* gene expression show opposing diurnal and circadian phases, i.e., *TH* is higher during the day and *TPH1* is higher at night, as well as opposing expression changes in long *vs* short photoperiod (Kang et al., 2007). Circadian rhythms and photoperiod also modulate the expression of clock genes in the turkey’s PMM (Leclerc et al., 2010).

The photoperiodic control of reproduction in birds is independent of melatonin and relies on the expression of photoreceptors in the mediobasal hypothalamus. These photoreceptors are responsive to different wavelengths, informing distinct light stimulation to brain sites associated with the control of seasonal reproduction (Nakane et al., 2010; Nakane and Yoshimura, 2019). For example, the turkey PMM expresses melanopsin (*OPN4* gene), a photoreceptor responsive to blue light (Kang et al., 2010). OPN4 expression is higher at night and decreases in response to light pulses during the photosensitive period (Kang et al., 2010). Thirty minutes of light exposure during the photosensitive period induces Fos-ir in PMM neurons and increases *GNRH1* mRNA expression (Thayananuphat et al., 2007a, 2007b).

Melanopsin expression has also been observed in the PMM TH cells of domesticated Pekin drakes (*Anas platyrhynchos*). In this species, white and blue (but not red) light induced expression of *FRA2* (an immediate early gene) in PMM TH cells (Haas et al., 2017). Male white-throated sparrows (*Zonotrichia albicollis*) kept in short-day lengths and exposed to a single long day showed change in the expression of another immediate early gene, *EGR1*, in dopaminergic neurons of the PMM. *EGR1* expression was delayed for about 11 h after the shift, still before the activation of GnRH neurons was detected. In this species, no daily rhythm was observed in PMM TH expression (Maney et al., 2020). It is worth noting that the differences in timing of relevant gene expression observed in distinct species may be associated with experimental variables such as dynamic changes in gene expression, differences in brain circuitry, and breeding strategy.

In Pekin drakes, long-duration white light caused gonadal recrudescence, i.e., gonadal “awakening” after a period of inactivity. Blue or red lights alone, however, were not sufficient to induce this effect. Because the PMM neurons are mostly responsive to blue lights, the authors concluded that, in Pekin drakes, light-induced activation of PMM is not required for gonadal recrudescence (Haas et al., 2017). Similarly, PMM lesions in turkey hens did not affect egg laying or gonadal regression following long-photoperiod exposure, indicating that the PMM is dispensable for the initiation or termination of the breeding season (Moore et al., 2018).

As it stands, the role of PMM in birds is inconclusive and requires independent analysis in distinct avian species with different social behaviors and reproductive strategies. However, it is attempted to speculate that from an evolutionary perspective, the avian premammillary nucleus is well positioned to sense relevant environmental cues (photoperiod) modulating species-specific neuroendocrine and behavioral responses.

## 8 Concluding remarks

Successful social behavior critically depends on the sensory information from the external environment. In macrosmatic animals, such as rodents with a large olfactory system and a highly developed sense of smell, detecting odorants is essential for interactions with conspecifics. These interactions include discriminating sexual mates and competitors and defending the territory and offspring. However, for microsmatic species, such as primates, how external cues are integrated into behavioral responses remains poorly understood, with limited knowledge about hypothalamic sites associated with social behaviors. In seasonal breeders, photoperiod or perceived day length plays a critical role in social behavior and reproductive success, indicating an evolutionary adaptation to environmental cues that align with species-specific reproductive strategies.

External environmental cues alone do not ensure successful behavior. The individual’s internal state is key in providing context for distinct social behaviors. Circulating hormones that indicate sex, reproductive status, and nutritional state are fundamental in facilitating behavioral choices. In most vertebrates, including primates, the hypothalamus integrates these external and internal cues.

In this review, we discussed the findings that position the hypothalamic PMv as a hub for integrating external and internal environmental cues. PMv neurons receive signals of conspecific odorants in rodents and of light in seasonal breeders, while also sensing sex hormones and metabolic cues, and projecting to neuroendocrine and behavioral effectors in the brain. Therefore, we propose that PMv neurons are key in defining context for behavioral output. However, much remains to be learned about the diverse strategies used by different species and the role played by the PMv in each of these social or species survival-oriented behaviors.

## References

[ref1] Ahima R. S. Dushay J. Flier S. N. Prabakaran D. Flier J. S. (1997). Leptin accelerates the onset of puberty in normal female mice. J. Clin. Invest. 99, 391–395. doi: 10.1172/JCI119172, PMID: 9022071 PMC507811

[ref2] Ahima R. S. Prabakaran D. Mantzoros C. Qu D. Lowell B. Maratos-Flier E.. (1996). Role of leptin in the neuroendocrine response to fasting. Nature 382, 250–252. doi: 10.1038/382250a0, PMID: 8717038

[ref3] Ahmadian-Moghadam H. Sadat-Shirazi M.-S. Zarrindast M.-R. (2018). Cocaine- and amphetamine-regulated transcript (CART): a multifaceted neuropeptide. Peptides 110, 56–77. doi: 10.1016/j.peptides.2018.10.008, PMID: 30391426

[ref4] Ahmed M. L. Ong K. K. Dunger D. B. (2009). Childhood obesity and the timing of puberty. Trends Endocrinol. Metabol. 20, 237–242. doi: 10.1016/j.tem.2009.02.004, PMID: 19541497

[ref5] Anderson D. J. (2012). Optogenetics, sex, and violence in the brain: implications for psychiatry. Biol. Psychiatry 71, 1081–1089. doi: 10.1016/j.biopsych.2011.11.012, PMID: 22209636 PMC3380604

[ref6] Anderson G. M. Hill J. W. Kaiser U. B. Navarro V. M. Ong K. K. Perry J. R. B.. (2024). Metabolic control of puberty: 60 years in the footsteps of Kennedy and Mitra’s seminal work. Nat. Rev. Endocrinol. 20, 111–123. doi: 10.1038/s41574-023-00919-z, PMID: 38049643 PMC10843588

[ref7] Bakken T. Kang S. W. Kosonsiriluk S. Kuwayama T. Chaiseha Y. El Halawani M. E. (2014). Differential roles of hypothalamic serotonin receptor subtypes in the regulation of prolactin secretion in the Turkey hen. Acta Histochem. 116, 131–137. doi: 10.1016/j.acthis.2013.06.002, PMID: 23886495

[ref8] Barash I. A. Cheung C. C. Weigle D. S. Ren H. Kabigting E. B. Kuijper J. L.. (1996). Leptin is a metabolic signal to the reproductive system. Endocrinology 137, 3144–3147. doi: 10.1210/endo.137.7.8770941, PMID: 8770941

[ref9] Barrett P. Ebling F. J. P. Schuhler S. Wilson D. Ross A. W. Warner A.. (2007). Hypothalamic thyroid hormone catabolism acts as a gatekeeper for the seasonal control of body weight and reproduction. Endocrinology 148, 3608–3617. doi: 10.1210/en.2007-0316, PMID: 17478556

[ref10] Bayless D. W. Davis C.-H. O. Yang R. Wei Y. de Andrade Carvalho V. M. Knoedler J. R.. (2023). A neural circuit for male sexual behavior and reward. Cell 186, 3862–3881.e28. doi: 10.1016/j.cell.2023.07.021, PMID: 37572660 PMC10615179

[ref11] Bedenbaugh M. N. Brener S. C. Maldonado J. Lippert R. N. Sweeney P. Cone R. D.. (2022). Organization of neural systems expressing melanocortin-3 receptors in the mouse brain: evidence for sexual dimorphism. J. Comp. Neurol. 530, 2835–2851. doi: 10.1002/cne.25379, PMID: 35770983 PMC9724692

[ref12] Bellefontaine N. Chachlaki K. Parkash J. Vanacker C. Colledge W. H. d’Anglemont de Tassigny X.. (2014). Leptin-dependent neuronal NO signaling in the preoptic hypothalamus facilitates reproduction. J. Clin. Invest. 124, 2550–2559. doi: 10.1172/JCI65928, PMID: 24812663 PMC4089460

[ref13] Beltramino C. Taleisnik S. (1978). Facilitatory and inhibitory effects of electrochemical stimulation of the amygdala on the release of luteinizing hormone. Brain Res. 144, 95–107. doi: 10.1016/0006-8993(78)90437-7, PMID: 565243

[ref14] Beltramino C. Taleisnik S. (1979). Effect of electrochemical stimulation in the olfactory bulbs on the release of gonadotropin hormones in rats. Neuroendocrinology 28, 320–328. doi: 10.1159/000122879, PMID: 571541

[ref15] Beltramino C. Taleisnik S. (1980). Dual action of electrochemical stimulation of the bed nucleus of the stria terminalis on the release of LH. Neuroendocrinology 30, 238–242. doi: 10.1159/000123007, PMID: 7189584

[ref16] Beltramino C. Taleisnik S. (1983). Release of LH in the female rat by olfactory stimuli. Effect of the removal of the vomeronasal organs or lesioning of the accessory olfactory bulbs. Neuroendocrinology 36, 53–58. doi: 10.1159/000123436, PMID: 6828208

[ref17] Beltramino C. Taleisnik S. (1985). Ventral premammillary nuclei mediate pheromonal-induced LH release stimuli in the rat. Neuroendocrinology 41, 119–124. doi: 10.1159/000124164, PMID: 4047330

[ref18] Biro F. M. Galvez M. P. Greenspan L. C. Succop P. A. Vangeepuram N. Pinney S. M.. (2010). Pubertal assessment method and baseline characteristics in a mixed longitudinal study of girls. Pediatrics 126, e583–e590. doi: 10.1542/peds.2009-3079, PMID: 20696727 PMC4460992

[ref19] Boehm U. (2006). The vomeronasal system in mice: from the nose to the hypothalamus- and back! Semin. Cell Dev. Biol. 17, 471–479. doi: 10.1016/j.semcdb.2006.04.013, PMID: 16765613

[ref20] Boehm U. Zou Z. Buck L. B. (2005). Feedback loops link odor and pheromone signaling with reproduction. Cell 123, 683–695. doi: 10.1016/j.cell.2005.09.027, PMID: 16290036

[ref21] Borges B. C. Garcia-Galiano D. da Silveira Cruz-Machado S. Han X. Gavrilina G. B. Saunders T. L.. (2017). Obesity-induced infertility in male mice is associated with disruption of Crisp4 expression and sperm fertilization capacity. Endocrinology 158, 2930–2943. doi: 10.1210/en.2017-00295, PMID: 28911169 PMC5659670

[ref22] Bronson F. H. Manning J. M. (1991). The energetic regulation of ovulation: a realistic role for body fat. Biol. Reprod. 44, 945–950. doi: 10.1095/biolreprod44.6.945, PMID: 1873394

[ref23] Brown L. L. Pasi S. Etgen A. M. (1996). Estrogen regulation of mu opioid receptor density in hypothalamic premammillary nuclei. Brain Res. 742, 347–351. doi: 10.1016/s0006-8993(96)01089-x, PMID: 9117417

[ref24] Burt Solorzano C. M. McCartney C. R. (2010). Obesity and the pubertal transition in girls and boys. Reproduction 140, 399–410. doi: 10.1530/REP-10-0119, PMID: 20802107 PMC2931339

[ref25] Canteras N. S. Simerly R. B. Swanson L. W. (1992). Projections of the ventral premammillary nucleus. J. Comp. Neurol. 324, 195–212. doi: 10.1002/cne.903240205, PMID: 1430329

[ref26] Cara A. L. Henson E. L. Beekly B. G. Elias C. F. (2021). Distribution of androgen receptor mRNA in the prepubertal male and female mouse brain. J. Neuroendocrinol. 33:e13063. doi: 10.1111/jne.13063, PMID: 34866263 PMC8711114

[ref27] Cara A. L. Myers M. G. Elias C. F. (2020). Lack of AR in LepRb cells disrupts ambulatory activity and neuroendocrine axes in a sex-specific manner in mice. Endocrinology 161:bqaa110. doi: 10.1210/endocr/bqaa110, PMID: 32609838 PMC7383963

[ref9001] Cara A. L. Burger L. L. Beekly B. G. Allen S. J. Henson E. L. Auchus R. J.. (2023). Deletion of androgen receptor in LepRb cells improves estrous cycles in prenatally androgenized mice. Endocrinology. 164:bqad015. doi: 10.1210/endocr/bqad015 36683455 PMC10091504

[ref28] Caron E. Sachot C. Prevot V. Bouret S. G. (2010). Distribution of leptin-sensitive cells in the postnatal and adult mouse brain. J. Comp. Neurol. 518, 459–476. doi: 10.1002/cne.22219, PMID: 20017211

[ref29] Casanueva F. F. Dieguez C. (1999). Neuroendocrine regulation and actions of leptin. Front. Neuroendocrinol. 20, 317–363. doi: 10.1006/frne.1999.0187, PMID: 10569281

[ref30] Cavalcante J. C. Bittencourt J. C. Elias C. F. (2006a). Female odors stimulate CART neurons in the ventral premammillary nucleus of male rats. Physiol. Behav. 88, 160–166. doi: 10.1016/j.physbeh.2006.03.032, PMID: 16687159

[ref31] Cavalcante J. C. Bittencourt J. C. Elias C. F. (2014). Distribution of the neuronal inputs to the ventral premammillary nucleus of male and female rats. Brain Res. 1582, 77–90. doi: 10.1016/j.brainres.2014.07.034, PMID: 25084037 PMC4242707

[ref32] Cavalcante J. C. Candido P. L. Sita L. V. Do Nascimento E. S. de Souza Cavalcante J. De Oliveira Costa M. S.. (2011). Comparative distribution of cocaine- and amphetamine-regulated transcript (CART) in the hypothalamus of the capuchin monkey (*Cebus apella*) and the common marmoset (*Callithrix jacchus*). Brain Res. 1425, 47–61. doi: 10.1016/j.brainres.2011.09.020, PMID: 22030409

[ref33] Cavalcante J. C. Sita L. V. Mascaro M. B. Bittencourt J. C. Elias C. F. (2006b). Distribution of urocortin 3 neurons innervating the ventral premammillary nucleus in the rat brain. Brain Res. 1089, 116–125. doi: 10.1016/j.brainres.2006.03.043, PMID: 16638605

[ref34] Chachlaki K. Messina A. Delli V. Leysen V. Maurnyi C. Huber C.. (2022). NOS1 mutations cause hypogonadotropic hypogonadism with sensory and cognitive deficits that can be reversed in infantile mice. Sci. Transl. Med. 14:eabh2369. doi: 10.1126/scitranslmed.abh2369, PMID: 36197968 PMC7613826

[ref35] Chamero P. Katsoulidou V. Hendrix P. Bufe B. Roberts R. Matsunami H.. (2011). G protein G(alpha)o is essential for vomeronasal function and aggressive behavior in mice. Proc. Natl. Acad. Sci. USA 108, 12898–12903. doi: 10.1073/pnas.1107770108, PMID: 21768373 PMC3150917

[ref36] Chehab F. F. Lim M. E. Lu R. (1996). Correction of the sterility defect in homozygous obese female mice by treatment with the human recombinant leptin. Nat. Genet. 12, 318–320. doi: 10.1038/ng0396-318, PMID: 8589726

[ref37] Chen A.-X. Yan J.-J. Zhang W. Wang L. Yu Z.-X. Ding X.-J.. (2020). Specific hypothalamic neurons required for sensing conspecific male cues relevant to inter-male aggression. Neuron 108, 763–774.e6. doi: 10.1016/j.neuron.2020.08.025, PMID: 32961129

[ref38] Cheung C. C. Thornton J. E. Kuijper J. L. Weigle D. S. Clifton D. K. Steiner R. A. (1997). Leptin is a metabolic gate for the onset of puberty in the female rat. Endocrinology 138, 855–858. doi: 10.1210/endo.138.2.5054, PMID: 9003028

[ref39] Childs G. V. Odle A. K. MacNicol M. C. MacNicol A. M. (2021). The importance of leptin to reproduction. Endocrinology 162:bqaa204. doi: 10.1210/endocr/bqaa204, PMID: 33165520 PMC7749705

[ref40] Ciccarelli A. Weijers D. Kwan W. Warner C. Bourne J. Gross C. T. (2021). Sexually dimorphic perineuronal nets in the rodent and primate reproductive circuit. J. Comp. Neurol. 529, 3274–3291. doi: 10.1002/cne.25167, PMID: 33950531

[ref41] Coolen L. M. Peters H. J. Veening J. G. (1996). Fos immunoreactivity in the rat brain following consummatory elements of sexual behavior: a sex comparison. Brain Res. 738, 67–82. doi: 10.1016/0006-8993(96)00763-9, PMID: 8949929

[ref42] Couceyro P. R. Koylu E. O. Kuhar M. J. (1997). Further studies on the anatomical distribution of CART by in situ hybridization. J. Chem. Neuroanat. 12, 229–241. doi: 10.1016/S0891-0618(97)00212-3, PMID: 9243343

[ref9002] Crosby E. C. Showers M. J. (1969). “Com-parative anatomy of the preoptic and hypothalamic areas” in The Hypothalamus. eds. Haymaker W. Anderson E. Nauta W. J. H. (Springfield, Illinois: CC Thomas).

[ref43] Dardente H. Wood S. Ebling F. Sáenz de Miera C. (2019). An integrative view of mammalian seasonal neuroendocrinology. J. Neuroendocrinol. 31:e12729. doi: 10.1111/jne.12729, PMID: 31059174

[ref44] de Almeida R. M. M. Ferreira A. Agrati D. (2014). Sensory, hormonal, and neural basis of maternal aggression in rodents. Curr. Top. Behav. Neurosci. 17, 111–130. doi: 10.1007/7854_2014_312, PMID: 24841427

[ref45] de Roux N. Genin E. Carel J.-C. Matsuda F. Chaussain J.-L. Milgrom E. (2003). Hypogonadotropic hypogonadism due to loss of function of the KiSS1-derived peptide receptor GPR54. Proc. Natl. Acad. Sci. USA 100, 10972–10976. doi: 10.1073/pnas.1834399100, PMID: 12944565 PMC196911

[ref46] Denroche H. C. Glavas M. M. Tudurí E. Karunakaran S. Quong W. L. Philippe M.. (2016). Disrupted leptin signaling in the lateral hypothalamus and ventral Premammillary nucleus alters insulin and glucagon secretion and protects against diet-induced obesity. Endocrinology 157, 2671–2685. doi: 10.1210/en.2015-1998, PMID: 27183315

[ref47] Donato J. Cravo R. M. Frazão R. Gautron L. Scott M. M. Lachey J.. (2011). Leptin’s effect on puberty in mice is relayed by the ventral premammillary nucleus and does not require signaling in Kiss1 neurons. J. Clin. Invest. 121, 355–368. doi: 10.1172/JCI45106, PMID: 21183787 PMC3007164

[ref48] Donato J. Frazao R. Fukuda M. Vianna C. R. Elias C. F. (2010a). Leptin induces phosphorylation of neuronal nitric oxide synthase in defined hypothalamic neurons. Endocrinology 151, 5415–5427. doi: 10.1210/en.2010-0651, PMID: 20881244 PMC2954713

[ref49] Donato J. Jr. Cavalcante J. C. Silva R. J. Teixeira A. S. Bittencourt J. C. Elias C. F. (2010b). Male and female odors induce Fos expression in chemically defined neuronal population. Physiol. Behav. 99, 67–77. doi: 10.1016/j.physbeh.2009.10.012, PMID: 19857504

[ref50] Donato J. Jr. Lee C. Ratra D. V. Franci C. R. Canteras N. S. Elias C. F. (2013). Lesions of the ventral premammillary nucleus disrupt the dynamic changes in Kiss1 and GnRH expression characteristic of the proestrus-estrus transition. Neuroscience 241, 67–79. doi: 10.1016/j.neuroscience.2013.03.013, PMID: 23518222 PMC3661013

[ref51] Donato J. Silva R. J. Sita L. V. Lee S. Lee C. Lacchini S.. (2009). The ventral Premammillary nucleus links fasting-induced changes in leptin levels and coordinated luteinizing hormone secretion. J. Neurosci. 29, 5240–5250. doi: 10.1523/jneurosci.0405-09.2009, PMID: 19386920 PMC2696192

[ref52] Douglass J. McKinzie A. A. Couceyro P. (1995). PCR differential display identifies a rat brain mRNA that is transcriptionally regulated by cocaine and amphetamine. J. Neurosci. 15, 2471–2481. doi: 10.1523/JNEUROSCI.15-03-02471.1995, PMID: 7891182 PMC6578117

[ref53] Dubois-Dauphin M. Theler J. M. Zaganidis N. Dominik W. Tribollet E. Pévet P.. (1991). Expression of vasopressin receptors in hamster hypothalamus is sexually dimorphic and dependent upon photoperiod. Proc. Natl. Acad. Sci. USA 88, 11163–11167. doi: 10.1073/pnas.88.24.11163, PMID: 1837144 PMC53094

[ref54] Dunger D. B. Ahmed M. L. Ong K. K. (2005). Effects of obesity on growth and puberty. Best Pract. Res. Clin. Endocrinol. Metab. 19, 375–390. doi: 10.1016/j.beem.2005.04.005 16150381

[ref55] Egan O. K. Inglis M. A. Anderson G. M. (2017). Leptin signaling in AgRP neurons modulates puberty onset and adult fertility in mice. J. Neurosci. 37, 3875–3886. doi: 10.1523/jneurosci.3138-16.2017, PMID: 28275162 PMC6596709

[ref56] Elias C. F. (2012). Leptin action in pubertal development: recent advances and unanswered questions. Trends Endocrinol. Metabol. 23, 9–15. doi: 10.1016/j.tem.2011.09.002, PMID: 21978495 PMC3251729

[ref57] Elias C. F. Kelly J. F. Lee C. E. Ahima R. S. Drucker D. J. Saper C. B.. (2000). Chemical characterization of leptin-activated neurons in the rat brain. J. Comp. Neurol. 423, 261–281. doi: 10.1002/1096-9861(20000724)423:2<261::AID-CNE6>3.0.CO;2-6, PMID: 10867658

[ref58] Elias C. F. Lee C. E. Kelly J. F. Ahima R. S. Kuhar M. Saper C. B.. (2001). Characterization of CART neurons in the rat and human hypothalamus. J. Comp. Neurol. 432, 1–19. doi: 10.1002/cne.1085, PMID: 11241374

[ref59] Elias C. F. Purohit D. (2013). Leptin signaling and circuits in puberty and fertility. Cell. Mol. Life Sci. 70, 841–862. doi: 10.1007/s00018-012-1095-1, PMID: 22851226 PMC3568469

[ref60] Elmquist J. K. Ahima R. S. Maratos-Flier E. Flier J. S. Saper C. B. (1997). Leptin activates neurons in ventrobasal hypothalamus and brainstem. Endocrinology 138, 839–842. doi: 10.1210/endo.138.2.5033, PMID: 9003024

[ref61] Elmquist J. K. Bjørbæk C. Ahima R. S. Flier J. S. Saper C. B. (1998). Distributions of leptin receptor mRNA isoforms in the rat brain. J. Comp. Neurol. 395, 535–547. doi: 10.1002/(SICI)1096-9861(19980615)395:4<535::AID-CNE9>3.0.CO;2-2, PMID: 9619505

[ref62] Elmquist J. K. Elias C. F. Saper C. B. (1999). From lesions to leptin: hypothalamic control of food intake and body weight. Neuron 22, 221–232. doi: 10.1016/s0896-6273(00)81084-3, PMID: 10069329

[ref63] Farooqi I. S. Jebb S. A. Langmack G. Lawrence E. Cheetham C. H. Prentice A. M.. (1999). Effects of recombinant leptin therapy in a child with congenital leptin deficiency. New Engl. J. Med. 341, 879–884. doi: 10.1056/NEJM199909163411204, PMID: 10486419

[ref64] Fawcett J. W. Oohashi T. Pizzorusso T. (2019). The roles of perineuronal nets and the perinodal extracellular matrix in neuronal function. Nat. Rev. Neurosci. 20, 451–465. doi: 10.1038/s41583-019-0196-3, PMID: 31263252

[ref65] Ferreira A. Hansen S. (1986). Sensory control of maternal aggression in *Rattus norvegicus*. J. Comp. Psychol. 100, 173–177. doi: 10.1037/0735-7036.100.2.173, PMID: 3720285

[ref66] Fox C. A. Ross L. R. Jacobson C. D. (1991). Ontogeny of cells containing estrogen receptor-like immunoreactivity in the Brazilian opossum brain. Brain Res. Dev. Brain Res. 63, 209–219. doi: 10.1016/0165-3806(91)90080-3, PMID: 1790590

[ref67] Frisch R. E. (1985). Fatness, menarche, and female fertility. Perspect. Biol. Med. 28, 611–633. doi: 10.1353/pbm.1985.0010, PMID: 4034365

[ref68] Garcia-Galiano D. Borges B. C. Donato J. Allen S. J. Bellefontaine N. Wang M.. (2017). PI3Kα inactivation in leptin receptor cells increases leptin sensitivity but disrupts growth and reproduction. JCI Insight 2:e96728. doi: 10.1172/jci.insight.96728, PMID: 29212950 PMC5752267

[ref69] Gautron L. Cravo R. M. Elmquist J. K. Elias C. F. (2013). Discrete melanocortin-sensitive neuroanatomical pathway linking the ventral premmamillary nucleus to the paraventricular hypothalamus. Neuroscience 240, 70–82. doi: 10.1016/j.neuroscience.2013.02.024, PMID: 23485805 PMC3661020

[ref70] Georgescu T. Khant Aung Z. Grattan D. R. Brown R. S. E. (2022). Prolactin-mediated restraint of maternal aggression in lactation. Proc. Natl. Acad. Sci. USA 119:e2116972119. doi: 10.1073/pnas.2116972119, PMID: 35131854 PMC8833212

[ref71] Glezer I. Bittencourt J. C. Rivest S. (2009). Neuronal expression of Cd36, Cd44, and Cd83 antigen transcripts maps to distinct and specific murine brain circuits. J. Comp. Neurol. 517, 906–924. doi: 10.1002/cne.22185, PMID: 19844997

[ref72] Gotti S. Sica M. Viglietti-Panzica C. Panzica G. (2005). Distribution of nitric oxide synthase immunoreactivity in the mouse brain. Microsc. Res. Techniq. 68, 13–35. doi: 10.1002/jemt.20219, PMID: 16208717

[ref73] Gruaz N. M. Lalaoui M. Pierroz D. D. Englaro P. Sizonenko P. C. Blum W. F.. (1998). Chronic administration of leptin into the lateral ventricle induces sexual maturation in severely food-restricted female rats. J. Neuroendocrinol. 10, 627–633. doi: 10.1046/j.1365-2826.1998.00247.x, PMID: 9725715

[ref74] Guillamón A. Segovia S. (1997). Sex differences in the vomeronasal system. Brain Res. Bull. 44, 377–382. doi: 10.1016/s0361-9230(97)00217-7 9370202

[ref75] Gurdjian E. S. (1927). The diencephalon of the albino rat. J. Comp. Neurol. 43, 1–114. doi: 10.1002/cne.900430102

[ref76] Gyurko R. Leupen S. Huang P. L. (2002). Deletion of exon 6 of the neuronal nitric oxide synthase gene in mice results in hypogonadism and infertility. Endocrinology 143, 2767–2774. doi: 10.1210/endo.143.7.8921, PMID: 12072412

[ref77] Haas R. Alenciks E. Meddle S. Fraley G. S. (2017). Expression of deep brain photoreceptors in the Pekin drake: a possible role in the maintenance of testicular function. Poult. Sci. 96, 2908–2919. doi: 10.3382/ps/pex037, PMID: 28339754 PMC5850723

[ref78] Hadeishi Y. Wood R. I. (1996). Nitric oxide synthase in mating behavior circuitry of male Syrian hamster brain. J. Neurobiol. 30, 480–492. doi: 10.1002/(SICI)1097-4695(199608)30:4<480::AID-NEU4>3.0.CO;2-#, PMID: 8844512

[ref79] Halpern M. Martínez-Marcos A. (2003). Structure and function of the vomeronasal system: an update. Prog. Neurobiol. 70, 245–318. doi: 10.1016/s0301-0082(03)00103-5, PMID: 12951145

[ref80] Han X. Burger L. L. Garcia-Galiano D. Sim S. Allen S. J. Olson D. P.. (2020). Hypothalamic and cell-specific transcriptomes unravel a dynamic neuropil remodeling in leptin-induced and typical pubertal transition in female mice. iScience 23:101563. doi: 10.1016/j.isci.2020.101563, PMID: 33083731 PMC7522126

[ref81] Hashikawa K. Hashikawa Y. Tremblay R. Zhang J. Feng J. E. Sabol A.. (2017). Esr1+ cells in the ventromedial hypothalamus control female aggression. Nat. Neurosci. 20, 1580–1590. doi: 10.1038/nn.4644, PMID: 28920934 PMC5953764

[ref82] Haymaker W. Anderson E. Nauta W. J. H. (1969). The hypothalamus. Charles C: Thomas.

[ref83] Hetherington A. W. Ranson S. W. (1940). Hypothalamic lesions and adiposity in the rat. Anat. Rec. 78, 149–172. doi: 10.1002/ar.1090780203

[ref84] Hill J. W. Elias C. F. (2018). Neuroanatomical framework of the metabolic control of reproduction. Physiol. Rev. 98, 2349–2380. doi: 10.1152/physrev.00033.2017, PMID: 30109817 PMC6170978

[ref85] Hope B. T. Michael G. J. Knigge K. M. Vincent S. R. (1991). Neuronal NADPH diaphorase is a nitric oxide synthase. Proc. Natl. Acad. Sci. USA 88, 2811–2814. doi: 10.1073/pnas.88.7.2811, PMID: 1707173 PMC51329

[ref86] Hyland L. Park S.-B. Abdelaziz Y. Abizaid A. (2021). Metabolic effects of ghrelin delivery into the hypothalamic ventral premammilary nucleus of male mice. Physiol. Behav. 228:113208. doi: 10.1016/j.physbeh.2020.113208, PMID: 33068562

[ref87] Itakura T. Murata K. Miyamichi K. Ishii K. K. Yoshihara Y. Touhara K. (2022). A single vomeronasal receptor promotes intermale aggression through dedicated hypothalamic neurons. Neuron 110, 2455–2469.e8. doi: 10.1016/j.neuron.2022.05.002, PMID: 35654036

[ref88] Jansen H. T. Lubbers L. S. Macchia E. DeGroot L. J. Lehman M. N. (1997). Thyroid hormone receptor (alpha) distribution in hamster and sheep brain: colocalization in gonadotropin-releasing hormone and other identified neurons. Endocrinology 138, 5039–5047. doi: 10.1210/endo.138.11.5481, PMID: 9348236

[ref89] Kameda Y. Miura M. Nishimaki T. (2001). Localization of neuropeptide Y mRNA and peptide in the chicken hypothalamus and their alterations after food deprivation, dehydration, and castration. J. Comp. Neurol. 436, 376–388. doi: 10.1002/cne.1074, PMID: 11438937

[ref90] Kang S. W. Leclerc B. Kosonsiriluk S. Mauro L. J. Iwasawa A. El Halawani M. E. (2010). Melanopsin expression in dopamine-melatonin neurons of the premammillary nucleus of the hypothalamus and seasonal reproduction in birds. Neuroscience 170, 200–213. doi: 10.1016/j.neuroscience.2010.06.082, PMID: 20620198

[ref91] Kang S. W. Leclerc B. Mauro L. J. El Halawani M. E. (2009). Serotonergic and catecholaminergic interactions with co-localised dopamine-melatonin neurones in the hypothalamus of the female Turkey. J. Neuroendocrinol. 21, 10–19. doi: 10.1111/j.1365-2826.2008.01804.x, PMID: 19094089

[ref92] Kang S. W. Thayananuphat A. Bakken T. El Halawani M. E. (2007). Dopamine-melatonin neurons in the avian hypothalamus controlling seasonal reproduction. Neuroscience 150, 223–233. doi: 10.1016/j.neuroscience.2007.08.031, PMID: 17935892

[ref93] Kappers C. U. A. Huber G. C. Crosby E. C. (1936). The comparative anatomy of the nervous system of vertebrates including man. J. Nerv. Ment. Dis. 84, 709–711. doi: 10.1097/00005053-193612000-00041

[ref94] Kohl J. Babayan B. M. Rubinstein N. D. Autry A. E. Marin-Rodriguez B. Kapoor V.. (2018). Functional circuit architecture underlying parental behaviour. Nature 556, 326–331. doi: 10.1038/s41586-018-0027-0, PMID: 29643503 PMC5908752

[ref95] Kollack-Walker S. Newman S. W. (1995). Mating and agonistic behavior produce different patterns of Fos immunolabeling in the male Syrian hamster brain. Neuroscience 66, 721–736. doi: 10.1016/0306-4522(94)00563-k, PMID: 7644033

[ref96] Koylu E. O. Couceyro P. R. Lambert P. D. Kuhar M. J. (1998). Cocaine- and amphetamine-regulated transcript peptide immunohistochemical localization in the rat brain. J. Comp. Neurol. 391, 115–132. doi: 10.1002/(SICI)1096-9861(19980202)391:1<115::AID-CNE10>3.0.CO;2-X, PMID: 9527537

[ref97] Koylu E. O. Couceyro P. R. Lambert P. D. Ling N. C. DeSouza E. B. Kuhar M. J. (1997). Immunohistochemical localization of novel CART peptides in rat hypothalamus, pituitary and adrenal gland. J. Neuroendocrinol. 9, 823–833. doi: 10.1046/j.1365-2826.1997.00651.x, PMID: 9419833

[ref98] Krieg W. J. S. (1932). The hypothalamus of the albino rat. J. Comp. Neurol. 55, 19–89. doi: 10.1002/cne.900550104

[ref99] Lane P. W. Dickie M. M. (1954). Fertile obese male mice. Relative sterility in obese males corrected by dietary restrictions. J. Hered. 45, 56–58. doi: 10.1093/oxfordjournals.jhered.a106439

[ref100] Lantos T. A. Görcs T. J. Palkovits M. (1995). Immunohistochemical mapping of neuropeptides in the premamillary region of the hypothalamus in rats. Brain Res. Brain Res. Rev. 20, 209–249. doi: 10.1016/0165-0173(94)00013-f, PMID: 7795657

[ref101] Larsen P. J. (1992). Distribution of substance P-immunoreactive elements in the preoptic area and the hypothalamus of the rat. J. Comp. Neurol. 316, 287–313. doi: 10.1002/cne.903160304, PMID: 1374435

[ref102] Leclerc B. Kang S. W. Mauro L. J. Kosonsiriluk S. Chaiseha Y. El Halawani M. E. (2010). Photoperiodic modulation of clock gene expression in the avian premammillary nucleus. J. Neuroendocrinol. 22, 119–128. doi: 10.1111/j.1365-2826.2009.01942.x, PMID: 20002961

[ref103] Leshan R. L. Greenwald-Yarnell M. Patterson C. M. Gonzalez I. E. Myers M. G. (2012). Leptin action through hypothalamic nitric oxide synthase-1-expressing neurons controls energy balance. Nat. Med. 18, 820–823. doi: 10.1038/nm.2724, PMID: 22522563 PMC3531967

[ref104] Leshan R. L. Louis G. W. Jo Y.-H. Rhodes C. J. Münzberg H. Myers M. G. (2009). Direct innervation of GnRH neurons by metabolic- and sexual odorant-sensing leptin receptor neurons in the hypothalamic ventral premammillary nucleus. J. Neurosci. 29, 3138–3147. doi: 10.1523/JNEUROSCI.0155-09.2009, PMID: 19279251 PMC2675106

[ref105] Leshan R. L. Pfaff D. W. (2014). The hypothalamic ventral premammillary nucleus: a key site in leptin’s regulation of reproduction. J. Chem. Neuroanat. 61-62, 239–247. doi: 10.1016/j.jchemneu.2014.08.008, PMID: 25172030

[ref106] Leslie R. A. Sanders S. J. K. Anderson S. I. Schuhler S. Horan T. L. Ebling F. J. P. (2001). Appositions between cocaine and amphetamine-related transcript- and gonadotropin releasing hormone-immunoreactive neurons in the hypothalamus of the Siberian hamster. Neurosci. Lett. 314, 111–114. doi: 10.1016/S0304-3940(01)02291-1, PMID: 11704296

[ref107] Lin D. Boyle M. P. Dollar P. Lee H. Lein E. S. Perona P.. (2011). Functional identification of an aggression locus in the mouse hypothalamus. Nature 470, 221–226. doi: 10.1038/nature09736, PMID: 21307935 PMC3075820

[ref108] Lin W. McKinney K. Liu L. Lakhlani S. Jennes L. (2003). Distribution of vesicular glutamate transporter-2 messenger ribonucleic acid and protein in the septum-hypothalamus of the rat. Endocrinology 144, 662–670. doi: 10.1210/en.2002-220908, PMID: 12538629

[ref109] Liu H. Kishi T. Roseberry A. G. Cai X. Lee C. E. Montez J. M.. (2003). Transgenic mice expressing green fluorescent protein under the control of the melanocortin-4 receptor promoter. J. Neurosci. 23, 7143–7154. doi: 10.1523/JNEUROSCI.23-18-07143.2003, PMID: 12904474 PMC6740648

[ref110] Loucks A. B. Verdun M. Heath E. M. (1998). Low energy availability, not stress of exercise, alters LH pulsatility in exercising women. J. Appl. Physiol. 84, 37–46. doi: 10.1152/jappl.1998.84.1.37, PMID: 9451615

[ref111] Louis G. W. Greenwald-Yarnell M. Phillips R. Coolen L. M. Lehman M. N. Myers M. G. (2011). Molecular mapping of the neural pathways linking leptin to the neuroendocrine reproductive axis. Endocrinology 152, 2302–2310. doi: 10.1210/en.2011-0096, PMID: 21427219 PMC3100610

[ref112] Lübke K. T. Pause B. M. (2015). Always follow your nose: the functional significance of social chemosignals in human reproduction and survival. Horm. Behav. 68, 134–144. doi: 10.1016/j.yhbeh.2014.10.001, PMID: 25637403

[ref113] Mahany E. B. Han X. Borges B. C. da Silveira Cruz-Machado S. Allen S. J. Garcia-Galiano D.. (2018). Obesity and high-fat diet induce distinct changes in placental gene expression and pregnancy outcome. Endocrinology 159, 1718–1733. doi: 10.1210/en.2017-03053, PMID: 29438518 PMC6456933

[ref114] Malpaux B. Daveau A. Maurice-Mandon F. Duarte G. Chemineau P. (1998). Evidence that melatonin acts in the premammillary hypothalamic area to control reproduction in the ewe: presence of binding sites and stimulation of luteinizing hormone secretion by in situ microimplant delivery. Endocrinology 139, 1508–1516. doi: 10.1210/endo.139.4.5879, PMID: 9528928

[ref115] Maney D. L. Aldredge R. A. Edwards S. H. A. James N. P. Sockman K. W. (2020). Time course of photo-induced Egr-1 expression in the hypothalamus of a seasonally breeding songbird. Mol. Cell. Endocrinol. 512:110854. doi: 10.1016/j.mce.2020.110854, PMID: 32422399 PMC7347413

[ref116] Marcus J. N. Aschkenasi C. J. Lee C. E. Chemelli R. M. Saper C. B. Yanagisawa M.. (2001). Differential expression of orexin receptors 1 and 2 in the rat brain. J. Comp. Neurol. 435, 6–25. doi: 10.1002/cne.1190, PMID: 11370008

[ref117] Marsteller F. A. Lynch C. B. (1987). Reproductive responses to variation in temperature and food supply by house mice. I. Mating and pregnancy. Biol. Reprod. 37, 838–843. doi: 10.1095/biolreprod37.4.838, PMID: 3689852

[ref118] Martin C. Navarro V. M. Simavli S. Vong L. Carroll R. S. Lowell B. B.. (2014). Leptin-responsive GABAergic neurons regulate fertility through pathways that result in reduced kisspeptinergic tone. J. Neurosci. 34, 6047–6056. doi: 10.1523/JNEUROSCI.3003-13.2014, PMID: 24760864 PMC3996222

[ref119] Martinez-Vargas M. C. Stumpf W. E. Sar M. (1976). Anatomical distribution of estrogen target cells in the avian CNS: a comparison with the mammalian CNS. J. Comp. Neurol. 167, 83–103. doi: 10.1002/cne.901670106, PMID: 1270623

[ref120] Meister B. Elde R. (1993). Dopamine transporter mRNA in neurons of the rat hypothalamus. Neuroendocrinology 58, 388–395, PMID: 8284024 10.1159/000126568

[ref121] Merchenthaler I. Lane M. V. Numan S. Dellovade T. L. (2004). Distribution of estrogen receptor alpha and beta in the mouse central nervous system: *in vivo* autoradiographic and immunocytochemical analyses. J. Comp. Neurol. 473, 270–291. doi: 10.1002/cne.20128, PMID: 15101093

[ref122] Merlino D. J. Barton J. R. Charsar B. A. Byrne M. D. Rappaport J. A. Smeyne R. J.. (2019). Two distinct GUCY2C circuits with PMV (hypothalamic) and SN/VTA (midbrain) origin. Brain Struct. Funct. 224, 2983–2999. doi: 10.1007/s00429-019-01949-y, PMID: 31485718 PMC6778723

[ref123] Michael R. P. Clancy A. N. Zumpe D. (1995). Distribution of androgen receptor-like immunoreactivity in the brains of cynomolgus monkeys. J. Neuroendocrinol. 7, 713–719. doi: 10.1111/j.1365-2826.1995.tb00813.x, PMID: 8547949

[ref124] Michael R. P. Rees H. D. (1982). Autoradiographic localization of 3H-dihydrotestosterone in the preoptic area, hypothalamus, and amygdala of a male rhesus monkey. Life Sci. 30, 2087–2093. doi: 10.1016/0024-3205(82)90450-7, PMID: 7109838

[ref125] Migaud M. Daveau A. Malpaux B. (2005). MTNR1A melatonin receptors in the ovine premammillary hypothalamus: day-night variation in the expression of the transcripts. Biol. Reprod. 72, 393–398. doi: 10.1095/biolreprod.104.030064, PMID: 15470001

[ref126] Montagnese C. M. Zachar G. Bálint E. Csillag A. (2008). Afferent connections of septal nuclei of the domestic chick (Gallus domesticus): a retrograde pathway tracing study. J. Comp. Neurol. 511, 109–150. doi: 10.1002/cne.21837, PMID: 18752269

[ref127] Moore A. F. Cassone V. M. Alloway K. D. Bartell P. A. (2018). The premammillary nucleus of the hypothalamus is not necessary for photoperiodic timekeeping in female turkeys (*Meleagris gallopavo*). PLoS One 13:e0190274. doi: 10.1371/journal.pone.0190274, PMID: 29462137 PMC5819771

[ref128] Motta S. C. Guimarães C. C. Furigo I. C. Sukikara M. H. Baldo M. V. C. Lonstein J. S.. (2013). Ventral premammillary nucleus as a critical sensory relay to the maternal aggression network. Proc. Natl. Acad. Sci. USA 110, 14438–14443. doi: 10.1073/pnas.1305581110, PMID: 23918394 PMC3761625

[ref129] Nagatani S. Guthikonda P. Thompson R. C. Tsukamura H. Maeda K. I. Foster D. L. (1998). Evidence for GnRH regulation by leptin: leptin administration prevents reduced pulsatile LH secretion during fasting. Neuroendocrinology 67, 370–376. doi: 10.1159/000054335, PMID: 9662716

[ref130] Nakane Y. Ikegami K. Ono H. Yamamoto N. Yoshida S. Hirunagi K.. (2010). A mammalian neural tissue opsin (opsin 5) is a deep brain photoreceptor in birds. Proc. Natl. Acad. Sci. USA 107, 15264–15268. doi: 10.1073/pnas.1006393107, PMID: 20679218 PMC2930557

[ref131] Nakane Y. Yoshimura T. (2019). Photoperiodic regulation of reproduction in vertebrates. Annu. Rev. Anim. Biosci. 7, 173–194. doi: 10.1146/annurev-animal-020518-115216, PMID: 30332291

[ref132] Nakano K. Suga S. Kondo Y. Sato T. Sakuma Y. (1997). Estrogen-excitable forebrain projections to the ventral premammillary nucleus of the female rat. Neurosci. Lett. 225, 17–20. doi: 10.1016/s0304-3940(97)00175-4, PMID: 9143007

[ref133] Pan L. Xiao S. Xu Z. Li W. Zhao L. Zhang L.. (2024). Orexin-a attenuated motion sickness through modulating neural activity in hypothalamus nuclei. Br. J. Pharmacol. 181, 1474–1493. doi: 10.1111/bph.16307, PMID: 38129941

[ref134] Papp R. S. Könczöl K. Sípos K. Tóth Z. E. (2025). Nesfatin-1 neurons in the ventral Premammillary nucleus integrate metabolic and reproductive signals in male rats. Int. J. Mol. Sci. 26:739. doi: 10.3390/ijms26020739, PMID: 39859453 PMC11765514

[ref135] Pirke K. M. Schweiger U. Lemmel W. Krieg J. C. Berger M. (1985). The influence of dieting on the menstrual cycle of healthy young women. J. Clin. Endocrinol. Metab. 60, 1174–1179. doi: 10.1210/jcem-60-6-1174, PMID: 3923022

[ref136] Ramón y Cajal S. (1911). Histology of the nervous system of man and vertebrates. English (reprinted in translation, N. Swanson and L. Swanson 1995). Oxford, UK: Oxford University Press.

[ref137] Reinehr T. Roth C. L. (2019). Is there a causal relationship between obesity and puberty? Lancet child Adolesc. Health 3, 44–54. doi: 10.1016/S2352-4642(18)30306-7, PMID: 30446301

[ref138] Reis M. E. M. D. Araújo L. T. F. Andrade W. M. G. Resende N. D. S. Lima R. R. M. Nascimento E. S.. (2018). Distribution of nitric oxide synthase in the rock cavy (*Kerodon rupestris*) brain I: the diencephalon. Brain Res. 1685, 60–78. doi: 10.1016/j.brainres.2018.01.020, PMID: 29438673

[ref139] Rodrigo J. Springall D. R. Uttenthal O. Bentura M. L. Abadia-Molina F. Riveros-Moreno V.. (1994). Localization of nitric oxide synthase in the adult rat brain. Philos. Trans. R. Soc. B Biol. Sci. 345, 175–221. doi: 10.1098/rstb.1994.0096, PMID: 7526408

[ref140] Rondini T. A. Baddini S. P. Sousa L. F. Bittencourt J. C. Elias C. F. (2004). Hypothalamic cocaine- and amphetamine-regulated transcript neurons project to areas expressing gonadotropin releasing hormone immunoreactivity and to the anteroventral periventricular nucleus in male and female rats. Neuroscience 125, 735–748. doi: 10.1016/j.neuroscience.2003.12.045, PMID: 15099687

[ref141] Rosenfield R. L. Lipton R. B. Drum M. L. (2009). Thelarche, pubarche, and menarche attainment in children with normal and elevated body mass index. Pediatrics 123, 84–88. doi: 10.1542/peds.2008-0146, PMID: 19117864

[ref142] Ross R. A. Leon S. Madara J. C. Schafer D. Fergani C. Maguire C. A.. (2018). PACAP neurons in the ventral premammillary nucleus regulate reproductive function in the female mouse. eLife 7:e35960. doi: 10.7554/eLife.35960, PMID: 29905528 PMC6013253

[ref143] Sáenz de Miera C. Bellefontaine N. Allen S. J. Myers M. G. Elias C. F. (2024). Glutamate neurotransmission from leptin receptor cells is required for typical puberty and reproductive function in female mice. eLife 13:RP93204. doi: 10.7554/eLife.93204, PMID: 39007235 PMC11249761

[ref144] Sáenz De Miera C. Bellefontaine N. Silveira M. A. Fortin C. N. Zampieri T. T. Donato J.. (2025). Nutritionally responsive PMv DAT neurons are dynamically regulated during pubertal transition. BioRxiv. doi: 10.1101/2025.02.03.636271, PMID: 39975315 PMC11838509

[ref145] Scalia F. Winans S. S. (1975). The differential projections of the olfactory bulb and accessory olfactory bulb in mammals. J. Comp. Neurol. 161, 31–55. doi: 10.1002/cne.901610105, PMID: 1133226

[ref146] Schneider J. E. Goldman M. D. Tang S. Bean B. Ji H. Friedman M. I. (1998). Leptin indirectly affects estrous cycles by increasing metabolic fuel oxidation. Horm. Behav. 33, 217–228. doi: 10.1006/hbeh.1998.1453, PMID: 9698504

[ref147] Scott M. M. Lachey J. L. Sternson S. M. Lee C. E. Elias C. F. Friedman J. M.. (2009). Leptin targets in the mouse brain. J. Comp. Neurol. 514, 518–532. doi: 10.1002/cne.22025, PMID: 19350671 PMC2710238

[ref148] Segovia S. Guillamón A. (1993). Sexual dimorphism in the vomeronasal pathway and sex differences in reproductive behaviors. Brain Res. Rev. 18, 51–74. doi: 10.1016/0165-0173(93)90007-m, PMID: 8467350

[ref149] Seminara S. B. Messager S. Chatzidaki E. E. Thresher R. R. Acierno J. S. Shagoury J. K.. (2003). The GPR54 gene as a regulator of puberty. New Engl. J. Med. 349, 1614–1627. doi: 10.1056/NEJMoa035322, PMID: 14573733

[ref150] Shimada S. Inagaki S. Kubota Y. Kito S. Shiotani Y. Tohyama M. (1987). Coexistence of substance P- and enkephalin-like peptides in single neurons of the rat hypothalamus. Brain Res. 425, 256–262. doi: 10.1016/0006-8993(87)90508-7, PMID: 2448005

[ref151] Shiraishi T. Mager M. (1980a). 2-deoxy-D-glucose-induced hypothermia: thermoregulatory pathways in rat. Am. J. Phys. 239, R270–R276. doi: 10.1152/ajpregu.1980.239.3.R270, PMID: 7435598

[ref152] Shiraishi T. Mager M. (1980b). Hypothermia following injection of 2-deoxy-D-glucose into selected hypothalamic sites. Am. J. Phys. 239, R265–R269. doi: 10.1152/ajpregu.1980.239.3.R265, PMID: 7435597

[ref153] Shughrue P. J. Merchenthaler I. (2001). Distribution of estrogen receptor beta immunoreactivity in the rat central nervous system. J. Comp. Neurol. 436, 64–81. doi: 10.1002/cne.1054 11413547

[ref154] Silveira M. A. Zampieri T. T. Furigo I. C. Abdulkader F. Donato J. Frazao R. (2019). Acute effects of somatomammotropin hormones on neuronal components of the hypothalamic-pituitary-gonadal axis. Brain Res. 1714, 210–217. doi: 10.1016/j.brainres.2019.03.003, PMID: 30851245

[ref155] Simerly R. B. Chang C. Muramatsu M. Swanson L. W. (1990). Distribution of androgen and estrogen receptor mRNA-containing cells in the rat brain: an in situ hybridization study. J. Comp. Neurol. 294, 76–95. doi: 10.1002/cne.902940107, PMID: 2324335

[ref156] Singh O. Agarwal N. Yadav A. Basu S. Malik S. Rani S.. (2020). Concurrent changes in photoperiod-induced seasonal phenotypes and hypothalamic CART peptide-containing systems in night-migratory redheaded buntings. Brain Struct. Funct. 225, 2775–2798. doi: 10.1007/s00429-020-02154-y, PMID: 33141294 PMC7608113

[ref157] Singh O. Kumar S. Singh U. Kumar V. Lechan R. M. Singru P. S. (2016). Cocaine- and amphetamine-regulated transcript peptide (CART) in the brain of zebra finch, *Taeniopygia guttata*: organization, interaction with neuropeptide Y, and response to changes in energy status. J. Comp. Neurol. 524, 3014–3041. doi: 10.1002/cne.24004, PMID: 27018984

[ref158] Sisk C. L. Zehr J. L. (2005). Pubertal hormones organize the adolescent brain and behavior. Front. Neuroendocrinol. 26, 163–174. doi: 10.1016/j.yfrne.2005.10.003, PMID: 16309736

[ref159] Sliwowska J. H. Billings H. J. Goodman R. L. Coolen L. M. Lehman M. N. (2004). The premammillary hypothalamic area of the ewe: anatomical characterization of a melatonin target area mediating seasonal reproduction. Biol. Reprod. 70, 1768–1775. doi: 10.1095/biolreprod.103.024182, PMID: 14973262

[ref160] Smolenski A. Burkhardt A. M. Eigenthaler M. Butt E. Gambaryan S. Lohmann S. M.. (1998). Functional analysis of cGMP-dependent protein kinases I and II as mediators of NO/cGMP effects. Naunyn Schmiedeberg's Arch. Pharmacol. 358, 134–139. doi: 10.1007/pl00005234, PMID: 9721015

[ref161] Soden M. E. Miller S. M. Burgeno L. M. Phillips P. E. M. Hnasko T. S. Zweifel L. S. (2016). Genetic isolation of hypothalamic neurons that regulate context-specific male social behavior. Cell Rep. 16, 304–313. doi: 10.1016/j.celrep.2016.05.067, PMID: 27346361 PMC4945459

[ref162] Sorg B. A. Berretta S. Blacktop J. M. Fawcett J. W. Kitagawa H. Kwok J. C. F.. (2016). Casting a wide net: role of Perineuronal nets in neural plasticity. J. Neurosci. 36, 11459–11468. doi: 10.1523/JNEUROSCI.2351-16.2016, PMID: 27911749 PMC5125213

[ref163] Spratt D. P. Herbison A. E. (2002). Projections of the sexually dimorphic calcitonin gene-related peptide neurons of the preoptic area determined by retrograde tracing in the female rat. J. Comp. Neurol. 445, 336–346. doi: 10.1002/cne.10195, PMID: 11920711

[ref164] Stagkourakis S. Spigolon G. Williams P. Protzmann J. Fisone G. Broberger C. (2018). A neural network for intermale aggression to establish social hierarchy. Nat. Neurosci. 21, 834–842. doi: 10.1038/s41593-018-0153-x, PMID: 29802391

[ref165] Stagkourakis S. Williams P. Spigolon G. Khanal S. Ziegler K. Heikkinen L.. (2024). Maternal aggression driven by the transient mobilisation of a dormant hormone-sensitive circuit. BioRxiv 2023:02.526862. doi: 10.1101/2023.02.02.526862, PMID: 38585740 PMC10996482

[ref166] Sternson S. M. Nicholas Betley J. Cao Z. F. H. (2013). Neural circuits and motivational processes for hunger. Curr. Opin. Neurobiol. 23, 353–360. doi: 10.1016/j.conb.2013.04.006, PMID: 23648085 PMC3948161

[ref167] Tena-Sempere M. Barreiro M. L. (2002). Leptin in male reproduction: the testis paradigm. Mol. Cell. Endocrinol. 188, 9–13. doi: 10.1016/s0303-7207(02)00008-4, PMID: 11911940

[ref168] Thayananuphat A. Kang S. W. Bakken T. Millam J. R. El Halawani M. E. (2007a). Rhythm-dependent light induction of the c-fos gene in the Turkey hypothalamus. J. Neuroendocrinol. 19, 407–417. doi: 10.1111/j.1365-2826.2007.01544.x, PMID: 17388817

[ref169] Thayananuphat A. Kang S. W. Bakken T. Millam J. R. El Halawani M. E. (2007b). Rhythmic dependent light induction of gonadotrophin-releasing hormone-I expression and activation of dopaminergic neurones within the premammillary nucleus of the Turkey hypothalamus. J. Neuroendocrinol. 19, 399–406. doi: 10.1111/j.1365-2826.2007.01545.x, PMID: 17388816

[ref170] Topaloglu A. K. Tello J. A. Kotan L. D. Ozbek M. N. Yilmaz M. B. Erdogan S.. (2012). Inactivating KISS1 mutation and hypogonadotropic hypogonadism. New Engl. J. Med. 366, 629–635. doi: 10.1056/NEJMoa1111184, PMID: 22335740

[ref171] van den Berg J. M. Ter Horst G. J. Koolhas J. M. (1983). The nucleo premammillaris ventralis (PMV) and aggressive behavior in the rat. Aggres. Behav. 9, 41–47.

[ref172] Veazey R. B. Amaral D. G. Cowan W. M. (1982). The morphology and connections of the posterior hypothalamus in the cynomolgus monkey (*Macaca fascicularis*). I. Cytoarchitectonic organization. J. Comp. Neurol. 207, 114–134. doi: 10.1002/cne.902070203, PMID: 6808030

[ref173] Veening J. G. Coolen L. M. de Jong T. R. Joosten H. W. de Boer S. F. Koolhaas J. M.. (2005). Do similar neural systems subserve aggressive and sexual behaviour in male rats? Insights from c-Fos and pharmacological studies. Eur. J. Pharmacol. 526, 226–239. doi: 10.1016/j.ejphar.2005.09.041, PMID: 16263109

[ref174] Venancio J. C. Margatho L. O. Rorato R. Rosales R. R. C. Debarba L. K. Coletti R.. (2017). Short-term high-fat diet increases leptin activation of CART neurons and advances puberty in female mice. Endocrinology 158, 3929–3942. doi: 10.1210/en.2017-00452, PMID: 28938405 PMC5695829

[ref175] Vincent S. R. Kimura H. (1992). Histochemical mapping of nitric oxide synthase in the rat brain. Neuroscience 46, 755–784. doi: 10.1016/0306-4522(92)90184-4, PMID: 1371855

[ref176] Wamsley J. K. Young W. S. Kuhar M. J. (1980). Immunohistochemical localization of enkephalin in rat forebrain. Brain Res. 190, 153–174. doi: 10.1016/0006-8993(80)91166-x, PMID: 6247008

[ref177] Wang X. Robinson P. J. (1997). Cyclic GMP-dependent protein kinase and cellular signaling in the nervous system. J. Neurochem. 68, 443–456. doi: 10.1046/j.1471-4159.1997.68020443.x, PMID: 9003029

[ref178] Wang L. Vanacker C. Burger L. L. Barnes T. Shah Y. M. Myers M. G.. (2019). Genetic dissection of the different roles of hypothalamic kisspeptin neurons in regulating female reproduction. eLife 8:e43999. doi: 10.7554/eLife.43999, PMID: 30946012 PMC6491090

[ref179] Wang Q. Yu K. Wang J. Lin H. Wu Y. Wang W. (2012). Predator stress-induced persistent emotional arousal is associated with alterations of plasma corticosterone and hippocampal steroid receptors in rat. Behav. Brain Res. 230, 167–174. doi: 10.1016/j.bbr.2012.01.051, PMID: 22327185

[ref180] Warden M. K. Young W. S. (1988). Distribution of cells containing mRNAs encoding substance P and neurokinin B in the rat central nervous system. J. Comp. Neurol. 272, 90–113. doi: 10.1002/cne.902720107, PMID: 2454979

[ref181] Warren M. P. Voussoughian F. Geer E. B. Hyle E. P. Adberg C. L. Ramos R. H. (1999). Functional hypothalamic amenorrhea: hypoleptinemia and disordered eating. J. Clin. Endocrinol. Metab. 84, 873–877. doi: 10.1210/jcem.84.3.5551, PMID: 10084564

[ref182] Wiegand S. J. Terasawa E. Bridson W. E. (1978). Persistent estrus and blockade of progesterone-induced LH release follows lesions which do not damage the suprachiasmatic nucleus. Endocrinology 102, 1645–1648. doi: 10.1210/endo-102-5-1645, PMID: 570487

[ref183] Williams K. W. Sohn J.-W. Donato J. Lee C. E. Zhao J. J. Elmquist J. K.. (2011). The acute effects of leptin require PI3K signaling in the hypothalamic ventral premammillary nucleus. J. Neurosci. 31, 13147–13156. doi: 10.1523/JNEUROSCI.2602-11.2011, PMID: 21917798 PMC3319415

[ref184] Wittmann G. Deli L. Kalló I. Hrabovszky E. Watanabe M. Liposits Z.. (2007). Distribution of type 1 cannabinoid receptor (CB1)-immunoreactive axons in the mouse hypothalamus. J. Comp. Neurol. 503, 270–279. doi: 10.1002/cne.21383, PMID: 17492633

[ref185] Wommack J. C. Delville Y. (2007). Stress, aggression, and puberty: neuroendocrine correlates of the development of agonistic behavior in Golden hamsters. Brain Behav. Evol. 70, 267–273. doi: 10.1159/000105490, PMID: 17914258

[ref186] Yip S. H. York J. Hyland B. Bunn S. J. Grattan D. R. (2018). Incomplete concordance of dopamine transporter Cre (DAT(IREScre))-mediated recombination and tyrosine hydroxylase immunoreactivity in the mouse forebrain. J. Chem. Neuroanat. 90, 40–48. doi: 10.1016/j.jchemneu.2017.12.002, PMID: 29217488

[ref187] Yokosuka M. Matsuoka M. Ohtani-Kaneko R. Iigo M. Hara M. Hirata K.. (1999). Female-soiled bedding induced fos immunoreactivity in the ventral part of the premammillary nucleus (PMv) of the male mouse. Physiol. Behav. 68, 257–261. doi: 10.1016/s0031-9384(99)00160-2, PMID: 10627089

[ref188] Yokosuka M. Prins G. S. Hayashi S. (1997). Co-localization of androgen receptor and nitric oxide synthase in the ventral premammillary nucleus of the newborn rat: an immunohistochemical study. Brain Res. Dev. Brain Res. 99, 226–233. doi: 10.1016/s0165-3806(96)00217-9, PMID: 9125476

[ref189] Yoon H. Enquist L. W. Dulac C. (2005). Olfactory inputs to hypothalamic neurons controlling reproduction and fertility. Cell 123, 669–682. doi: 10.1016/j.cell.2005.08.039, PMID: 16290037

[ref190] Yu W. H. Walczewska A. Karanth S. McCann S. M. (1997). Nitric oxide mediates leptin-induced luteinizing hormone-releasing hormone (LHRH) and LHRH and leptin-induced LH release from the pituitary gland. Endocrinology 138, 5055–5058. doi: 10.1210/endo.138.11.5649, PMID: 9348239

[ref191] Yuan X.-S. Xiang Z. Jiang J.-B. Yuan F. Zhang M.-T. Zhang K.-Y.. (2024). Leptin receptor neurons in the ventral premammillary nucleus modulate emotion-induced insomnia. Cell Discov. 10:59. doi: 10.1038/s41421-024-00676-x, PMID: 38830876 PMC11148181

[ref192] Ziegler D. R. Cullinan W. E. Herman J. P. (2002). Distribution of vesicular glutamate transporter mRNA in rat hypothalamus. J. Comp. Neurol. 448, 217–229. doi: 10.1002/cne.10257, PMID: 12115705

[ref193] Zigman J. M. Jones J. E. Lee C. E. Saper C. B. Elmquist J. K. (2006). Expression of ghrelin receptor mRNA in the rat and the mouse brain. J. Comp. Neurol. 494, 528–548. doi: 10.1002/cne.20823, PMID: 16320257 PMC4524499

[ref194] Zoli M. Agnati L. F. Tinner B. Steinbusch H. W. Fuxe K. (1993). Distribution of dopamine-immunoreactive neurons and their relationships to transmitter and hypothalamic hormone-immunoreactive neuronal systems in the rat mediobasal hypothalamus. A morphometric and microdensitometric analysis. J. Chem. Neuroanat. 6, 293–310. doi: 10.1016/0891-0618(93)90034-2, PMID: 7506039

[ref195] Zuure W. A. Roberts A. L. Quennell J. H. Anderson G. M. (2013). Leptin signaling in GABA neurons, but not glutamate neurons, is required for reproductive function. J. Neurosci. 33, 17874–17883. doi: 10.1523/JNEUROSCI.2278-13.2013, PMID: 24198376 PMC6618430

